# Top3α is the replicative topoisomerase in mitochondrial DNA replication

**DOI:** 10.1093/nar/gkac660

**Published:** 2022-07-29

**Authors:** Anu Hangas, Nina J Kekäläinen, Alisa Potter, Craig Michell, Kauko J Aho, Chiara Rutanen, Johannes N Spelbrink, Jaakko L Pohjoismäki, Steffi Goffart

**Affiliations:** Department of Environmental and Biological Sciences, University of Eastern Finland, PO Box 111, 80101 Joensuu, Finland; Department of Environmental and Biological Sciences, University of Eastern Finland, PO Box 111, 80101 Joensuu, Finland; Department of Environmental and Biological Sciences, University of Eastern Finland, PO Box 111, 80101 Joensuu, Finland; Radboud Center for Mitochondrial Medicine, Department of Paediatrics, Radboudumc, Nijmegen, The Netherlands; Department of Environmental and Biological Sciences, University of Eastern Finland, PO Box 111, 80101 Joensuu, Finland; Department of Environmental and Biological Sciences, University of Eastern Finland, PO Box 111, 80101 Joensuu, Finland; Department of Environmental and Biological Sciences, University of Eastern Finland, PO Box 111, 80101 Joensuu, Finland; Radboud Center for Mitochondrial Medicine, Department of Paediatrics, Radboudumc, Nijmegen, The Netherlands; Department of Environmental and Biological Sciences, University of Eastern Finland, PO Box 111, 80101 Joensuu, Finland; Department of Environmental and Biological Sciences, University of Eastern Finland, PO Box 111, 80101 Joensuu, Finland

## Abstract

Mitochondrial DNA has been investigated for nearly fifty years, but many aspects of the maintenance of this essential small genome remain unknown. Like any genome, mammalian mitochondrial DNA requires the function of topoisomerases to counter and regulate the topological tension arising during replication, transcription, segregation, and repair. However, the functions of the different mitochondrial topoisomerases are poorly understood. Here, we investigate the role of Topoisomerase 3α (Top3α) in mtDNA replication and transcription, providing evidence that this enzyme, previously reported to act in mtDNA segregation, also participates in mtDNA replication fork progression. Top3α knockdown caused replication fork stalling, increased mtDNA catenation and decreased mtDNA levels. Overexpression in contrast induced abundant double-strand breaks around the replication origin O_H_ and abortion of early replication, while at the same time improving the resolution of mtDNA replication termination intermediates. Both Top3α knockdown and overexpression affected mitochondrial RNA transcription, leading to a decrease in steady-state levels of mitochondrial transcripts. Together, our results indicate that the mitochondrial isoform of Top3α is not only involved in mtDNA segregation, as reported previously, but also supports the progression of the replication fork. Mitochondrial Top3α is also influencing the progression of transcription, with its absence affecting downstream transcript levels.

## INTRODUCTION

Topoisomerases are DNA-modifying enzymes that change the structure of DNA by catalyzing a temporary cut of the phosphodiester backbone, followed by a controlled modification of the topology and resealing of the strand break (1). The action of topoisomerases is required for nearly any process of DNA maintenance and expression (2). As with all genomes, mitochondrial DNA (mtDNA), the small circular DNA molecule essential for mitochondrial function, requires topoisomerases during replication, transcription, segregation and possibly repair. Although four different topoisomerases have been found in mammalian mitochondria (3), their precise function as well as their distribution of labor are yet unclear.

The only exclusively mitochondrial topoisomerase, the type 1B topoisomerase Top1mt, is able to relax both negative and positive supercoils on a DNA molecule through the induction of single-strand breaks (4). Fittingly, cells lacking Top1mt exhibit increased negative supercoiling of mtDNA (5), and although Top1mt (−/−) cells are viable, they possess a decreased ability to cope with genotoxic stress (6,7). Mitochondrial Topoisomerase 2 (Top2) exists in two isoforms, that are likely identical to the nuclear enzymes Top2α and Top2β (5). Top2α is expressed only in proliferating cells, and therefore mtDNA maintenance in differentiated cells depends on the action of Top2β. Poisoning of Top2 or knockdown of Top2β induces accumulation of positively supercoiled mtDNA molecules and inhibits initiation of replication (8).

The fourth mitochondrial topoisomerase, the mitochondrial isoform of Top3α, is transcribed from the same gene that also encodes the nuclear Top3α, using an alternative upstream start codon creating a mitochondrial localization signal at the N-terminal part of the protein (9). As this localization signal is removed upon protein import, the resulting functional protein is virtually identical to its nuclear counterpart. The fact that the same gene encodes both nuclear and mitochondrial Top3α has hindered the investigation of its mitochondrial function, e.g. knockout of Top3α in mice leads to early embryonic lethality (10). In the nucleus, Top3α interacts with the Bloom syndrome complex, resolving double-Holliday junctions during recombination. Impairment of this function by mutations in Top3α lead to chromosomal segregation defects and developmental disturbances (11). The mitochondrial role of Top3α was first studied in *Drosophila melanogaster*, where the homologue TopIII is essential for mtDNA maintenance (12). Recently, Nicholls *et al.* (13) provided evidence that in mitochondria mtTop3α is also essential for genome segregation, as the separation of newly replicated mtDNA is impaired and multi-genome aggregates accumulate upon silencing of Top3α expression.

Mitochondrial genome maintenance requires topological regulation not only for replication initiation and segregation. The progression of both the replication and transcription machineries cause the build-up of positive and negative supercoils, that need to be removed by a topoisomerase (14,15). Additionally, there is evidence for mitochondrial DNA recombination under stress conditions (16–19), which would likely require the participation of a topoisomerase in the resolution of crossover structures.

In this study, we aimed to investigate whether mtTop3α participates in replication fork progression or recombination of mitochondrial DNA, in analogy to its nuclear functions. For this, we employed siRNA-mediated knockdown of Top3α as well as the reciprocal approach using overexpression. To investigate the function of mitochondrial Top3α independently from its nuclear function, we modified the human Top3α coding sequence to achieve the overexpression of a strictly mitochondrially targeted version (mtTop3α) as well as a mitochondrially targeted catalytically deficient enzyme (mtTop3α-Y362F).

Both Top3α knockdown and excess of functional mtTop3α caused clear replication stalling on all regions of mtDNA. We found Top3α to localize in close proximity to the replication fork, corroborating a function in topology regulation during replication. Interestingly, the increased activity of mtTop3α, an enzyme able to create only single-strand breaks, caused high levels of double-strand breaks in the non-coding region of mtDNA, suggesting that the protein decatenates mtDNA using a single-stranded phase at the end of replication, in analogy to the function of TopIII in bacteria (20).

The manipulation of mtTop3α levels also affected mitochondrial transcript levels, suggesting a role in topology control during transcription or an indirect effect through the disturbance of replication. However, a potential function of mtTop3α during mtDNA recombination remains elusive.

Our findings show Top3α to be involved in topology regulation during replication fork progression and offer a more precise insight into the decatenating mechanism of mtDNA in the termination phase of replication.

## MATERIALS AND METHODS

### Creation of expression constructs

The human Top3α coding sequence (GenBank accession # NM_004618) including the mitochondrial targeting sequence and a C-terminal myc-tag, was cloned into pcDNA5 FRT/TO. The AUG of the nuclear Top3α isoform was mutated to GCG to enforce the exclusive expression of the mitochondrially targeted isoform. To enhance the mitochondrial localization of the resulting protein the nuclear localization signal at the C-terminal end was destroyed by mutating the bases at position 3207–3211 from AAAAG to CGACG. The resulting MTS-Top3α-NLSmut-myc (hereafter mtTop3α) construct therefore differs from the expected wild-type protein by K965A and R966A mutations, which do not have functional consequences for the enzymatic activity of the recombinant protein. The catalytically inactive version MTS-Top3α-NLSmut-Catmut-myc (hereafter mtTop3α-Y362F) was created by the replacement of the catalytic tyrosine at position 362 with phenylalanine (TAT > TTT at nts 1398–1390). To identify any nuclear effects of Top3α overexpression, a strictly nuclear version of Top3α lacking the mitochondrial targeting sequence (amino acids 1–22 of mtTop3α) with a C-terminal flag-tag was employed.

Human Top2β equipped with the mitochondrial targeting sequence of human cytochrome C as well as the empty pcDNA 5 FRT/TO vector were employed as controls, where indicated in the results. For BioID labelling the MTS-Top3α-NLS coding sequence was cloned into the MCS-BioID2-HA vector (21) by In-Fusion cloning (TaKaRa Biotech), and the resulting fusion gene containing the mitochondrially targeted Top3α-NLS, the BioID2 gene and a C-terminal HA-tag recloned into pcDNA5 FRT/TO. As control for the biotin pull-down assays, we also created a construct containing the mitochondrial targeting sequence of Top3α directly fused to the BioID2-HA coding sequence.

### Cell culture

Stable, inducible HEK293 and HeLa T-REx cells were created by Flp-recombination using the constructs described above or the empty pcDNA5 FRT/TO vector and the Flp-recombinase expression vector pOG44, according to the manufacturer's recommendations (Invitrogen). The established cell lines were cultured in Dulbecco's Modified Eagle Medium supplemented with 10% FBS (Gibco), 50 μg Hygromycin and 5 μg Blasticidin at 37°C in 8.5% CO_2_. Expression of the respective transgene was achieved by addition of 1 or 5 ng/ml doxycycline to the growth medium for the indicated time. siRNA knockdown and immunocytochemistry were performed in non-transgenic HeLa cells, grown under the same conditions, but without Hygromycin and Blasticidin.

### Extraction of mitochondria

Mitochondria were extracted from cultured cells using differential centrifugation and purification by sucrose gradient (22). The purified mitochondria were used for protein and nucleic acid extractions as well as BioID streptavidin purification of biotinylated proteins. For Chromatin immunoprecipitation only differential centrifugation was used.

### Protein extraction and western blots

Proteins were extracted from purified mitochondria using TOTEX buffer, separated over 8% Tris/glycine or 4–12% Tris-tricine SDS-PAGE, transferred to nitrocellulose membrane, incubated with antibodies (Table 1) as previously described (23) and ECL-exposed onto a Chemidoc imaging system (Bio-Rad Laboratories).

**Table 1.** Antibodies used in this study

Antibody	Dilution	Source	Identifier
Rabbit-anti-Top3α	1:4000	ProteinTech	14525-1-AP
Rabbit-anti-TWNK (PEO1)	1:1000	Elabscience	EAP1298
Rabbit-anti-POLG	1:1000	Abcam	Ab128899
Rabbit-anti-PolRmt	1:250	Abcam	Ab32988
Mouse-anti-HSP60	1:20 000	Antibodies-online	ABIN361784
Mouse-anti-vinculin	1:10 000	Sigma	V9264
Rabbit-anti-TOMM20	1:4000	Sigma	HPA011562
Rabbit-anti-MGME1	1:1000	Sigma	HPA040913
Rabbit-anti-Top2β	1:1000	Abcam	ab15565
Rabbit-anti-TFAM	1:1000	Aviva	ARP31400
Mouse-anti-β-tubulin	1:10 000	ProteinTech	66240-1-ig
Goat-anti-mouse IgG HRP	1:10 000	Antibodies-online	ABIN101744
Goat-anti-rabbit IgG HRP	1:15 000	Life Technologies	A16104
Mouse-anti-myc tag (ICC)	1:400	Tonbo	7-6784-U100
Rabbit-anti-TOMM20 (ICC)	1:400	Sigma	HPA011562
Rabbit-anti-mtSSB (ICC)	1:400	ProteinTech	122121-1-AP
Rabbit-anti-flag (ICC)	1:400	ProteinTech	20543
Mouse-anti-HSP60 (ICC)	1:400	Antibodies-online	ABIN361784
AlexaFluor 594 Goat-anti-rabbit IgG (ICC)	1:1000	Invitrogen	A11037
AlexaFluor 488 Goat-anti-mouse IgG (ICC)	1:1000	Invitrogen	A11029
AlexaFluor 594 Goat-anti-mouse IgG (ICC)	1:1000	Invitrogen	A11032
Streptavidin-Alexa350 conjugate	1:1000	Invitrogen	S11249

### Transient transfection and immunocytochemistry

For immunochemical detection of the various Top3α constructs HeLa cells were seeded onto glass coverslips and transiently transfected with the pcDNA constructs described above using Turbofect transfection reagent (Thermo), following the manufacturer's instruction. One day after transfection the cells were fixed with 3.3% PFA for 25 min at room temperature and permeabilized in PBS + 0.5 % Triton-X, 10% FBS for 15 min. The slides were incubated with primary antibodies in PBS + 0.1% Triton, 10% FBS for 1 h, washed in PBS, incubated with secondary antibodies for 1 h. After washing the cells were mounted and the fluorescent staining analyzed with a Zeiss Axioplan2 fluorescent microscope equipped with an Axiocam camera.

To judge the effect of Top3α expression on the mitochondrial network, HeLa TREx mtTop3α cells and control cells containing only the empty pcDNA5 FRT/TO vector were induced with 5 ng/ml doxycycline for 24 h and immunostained as described above using an antibody against TOMM20 and mtSSB. Pictures were taken with a Zeiss AxioObserver Z1 with an Axiocam camera.

Nascent transcripts were labelled in Top3α knockdown cells and controls by a 2 h incubation with 100 μM Ethynyl Uridine (EU). Cells were fixed and EU-containing RNA click-labelled with biotin-Dde-biotin picolylazide as described (24). Biotinylated nascent RNA was visualized using Streptavidin-350 and the mitochondrial network using an anti-HSP60-antibody and an anti-rabbit-Alexa595 secondary antibody. Pictures were taken with a Zeiss AxioObserver Z1 with an Axiocam camera and the EU signal colocalizing with the mitochondrial HSP60 staining was quantified for each condition from six pictures (100–150 cells) using Zen 2.0 software.

### siRNA knockdown

For knockdown of Top3α, HEK293 cells were transfected in 6-well plates with 25 pmol control siRNA (Silencer Select neg. control #1, Thermo #4390843) or 12.5 + 12.5 pmol for combined transfections with two siRNAs (Ambion Silencer Select ID s14310 and s14312) using Lipofectamine RNAiMAX reagent (Thermo) and following the manufacturer's instructions. To verify the specificity of the siRNA effects, a Dharmacon siGENOME human TOP3A smartpool (7156) was used at a concentration of 100 pmol per 6-well with similar results. For 2D analysis of knockdown effects in HeLa cells the transfection was repeated after three days, and total DNA extracted on day 6.

### Nucleic acid extraction

Total cell DNA was extracted as previously described (22), using proteinase K digest, phenol:chloroform extraction and ethanol precipitation. DNA samples were dissolved in TE buffer and digested with *Bgl*II to facilitate solubilization. Mitochondrial DNA was extracted similarly from isolated, sucrose-purified mitochondria, but not digested and instead directly dissolved in 20 mM HEPES, pH 7.4. RNA was extracted with TriReagent (Sigma) and analyzed by Northern blot as described (23). Mitochondrial RNA levels were quantified using probes against ND2 (nts 4470–5511 of human mtDNA), COXI (nts 6573–7038), ATP6 (nts 8562–9147), ND5 (13 641–13 777) and ND6 (nts 14 374–14 595) and normalized against 18S rRNA (nts 850-1347 bp of accession number NR_0032862) as a loading control.

### MtDNA copy number determination

Mitochondrial copy number per cell was determined from total DNA samples using quantitative PCR as previously described (23). For human mtDNA the primers HSmtDNA13456F (5′-ACC ATT GGC AGC CTA GCA TT-3′) and HSmtDNA13593R (5′-TGT CAG GGA GGT AGC GAT GA-3′) and the probe HSmtDNA13546F (5′-FAM-ACA AAC GCC TGA GCC CTA-MGBNFQ-3′) were used, for the nuclear gene APP the forward primer HS-APP- F (5′-TTT TTG TGT GCT CTC CCA GGT CT-3′), the reverse primer HS APP-R (5′-TGGTCACTGGTTGGTTGGC-3′) and the probe HS-APP (5′-VIC-CCC TGA ACT GCA GAT CAC CAA TGT GGT AG-MGBNFQ-3′) was used.

### Topology analysis

Topological forms of mtDNA were analyzed by agarose gel electrophoresis and Southern blotting (8). In brief, 2 μg of total DNA was separated over a 0.4% agarose gel in TBE, blotted and probed against nts 37–611 of human mtDNA. The identity of the various topological forms of mtDNA was investigated by treating the samples with T7 endonuclease (Thermo Scientific), *Escherichia coli* Topo I (New England Biolabs) or *E. coli* TopoIV (Inspiralis, Cat. No. T4001). 850 ng total cellular DNA was incubated for 30 min at 37°C in 20 μl 1× Cutsmart buffer with 10 U Topo 1, or in 20 μl TopoIV buffer with 10 U T7 endo, TopoIV or both. The reaction was stopped by addition of 5 μl DNA loading dye (10 mM Tris–HCl pH 7.6, 0.03 % bromophenol blue, 0.03% xylene cyanol FF, 60% glycerol, 60 mM EDTA), separated over a topology gel and blotted and probed as described above.

### 7S DNA quantification

Mitochondrial 7S DNA levels per mtDNA were quantified by Southern blotting using 2 μg total DNA digested with HindIII and heated for 10 min at 65°C and probed against nts 16 177–40 of human mtDNA and nts 30–525 of the human 28S coding sequence. 7S DNA, full-length mtDNA and 28S gene signal were quantified by phosphor storage screens using phosphorimager and the ratio of 7S per mtDNA as well as 7S per 18S signal calculated.

### Neutral/Neutral two-dimensional agarose gel electrophoresis

For two-dimensional agarose gels (2D-AGE), 5 μg of mtDNA or 10 μg of total DNA were digested with the indicated enzymes (Fastdigest, ThermoScientific), separated by two-dimensional agarose gel electrophoresis and blotted as described (25). The membranes were probed with ^32^P-labelled probes (nts 35–611 for HincII, PvuII and BamHI, nts 14 374–14 595 for DraI, nts 11 180–11 620 for *Bcl*I, nts 3601–4079 for *Acc*I) and exposed to phosphor storage screen or Kodax MS film.

### BioID2 affinity purification and identification by peptide mass fingerprinting

Six 15 cm plates of 293T-REx cells carrying the mitochondrially targeted MTS-BioID2-HA gene, the MTS-Top3α-BioID2-HA gene were induced for 24 h with 3 ng/ml doxycycline to achieve a low level of protein expression. The medium was changed to replace the doxycycline by 50 μM biotin and the cells incubated for another 24 h. Mitochondria were isolated by differential centrifugation and sucrose gradient purification as described above. The mitochondrial pellet was resuspended in 1 ml 50 mM Tris pH 7.5, 500 mM NaCl, 0.4% SDS, 1 mM DTT and 100 μl 20% Triton X-100 added to lyse the mitochondria completely. The lysate was diluted with 1 vol. of 50 mM Tris–HCl pH 7.5 and sonicated for 15 s at 50% amplitude and 0.5 s cycle on interval. Insoluble matter was removed by centrifugation (16 000 g 10 min 4°C) and the lysate rotated with 100 ul Streptavidin-beads (Invitrogen Dynabeads MyOne Strepavidin C1, #65001) overnight at 4°C. The magnetic beads were isolated, washed four times with 1 ml 50 mM Tris–HCl pH 7.4, 8 M Urea and once with 50 mM Tris–HCl pH 7.5. The bound biotinylated proteins were eluted with 60 μl 1× SDS-sample buffer and heating for 5 min at 98°C. Input, flow-through, wash samples and eluate were separated over a Bolt 4-20% Bis-Tris SDS gel (Invitrogen) and analyzed by western blot using antibodies against TWNK, TFAM, MGME1, Top2β, MRE11 and POLG.

To further identify proteins labelled by the mtTop3α-BioID2 fusion protein, biotinylated proteins from 1 mg isolated mitochondrial proteins were captured on Streptavidin beads for 4 h and washed as described above. The bound proteins were identified by Mass spectrometry analysis as described in Hensen *et al.* (26). In brief, the captured biotinylated proteins from three biological replicates of mtTop3α-BioID2 and MTS-BioID2 experiments were subjected to on-beads digestion with 1 μg of LysC (Wako, 125-02543) for 3 h at room temperature followed by overnight digestion with 1 μg of Trypsin (Promega, V511A) at +37°C. The resulting peptides were purified by Pierce Detergent Removal Spin Columns (Thermo Scientific, 87777), desalted with home-made C18 stage-tips, and analyzed by LC–MS/MS in duplicates using 35% of total sample volume per injection. MaxQuant v.1.6.10.43 software was used to match tryptic peptides to the human Uniprot database (ID UP000005640, release date 20210428) and to quantify identified proteins by the label-free quantification (LFQ) method as described in (26).

For the data analysis, averaged LFQ values of two technical replicates were used. Only proteins that were identified in at least two biological replicates of mtTop3α-BioID2 purification were included in the data analysis. LFQ values were log_2_-transformed and missing values were replaced by random values drawn from normal distribution in Perseus v.1.6.15.0 (27), using width = 0.3 and downshift = 1.8. The log_2_ fold change values (log_2_FC) were calculated as difference between mtTop3α-BioID2 and MTS-BioID2 log_2_-transformed averaged triplicates. Statistical significance of log_2_-transformed LFQ values was determined using unpaired two-sample Student's t-test and delimited by the *P*-value threshold of <0.05 and the log_2_FC threshold of $ \ge$1.5. For data visualization, negative log_10_-transformed p-values were plotted against log_2_FC of Top3α-BioID2 and MTS-BioID2.

### Chromatin immunoprecipitation

For chromatin immunoprecipitation 293 TRex mtTop3α-myc and mtTop3α-flag were induced for 12 h with 1 ng/ml doxycycline. Crude mitochondria isolated by differential centrifugation were crosslinked with 1% formaldehyde in isotonic homogenization buffer for 10 min at room temperature. The crosslinking was stopped by addition of 125 mM glycine for 5 min and centrifugation at 800 g for 5 min. The mitochondrial pellets were lyzed in ChIP buffer (25 mM HEPES–KOH (pH 7.6), 10 % glycerol, 5 mM MgCl_2_, 0.5 mM EDTA, 0.5% Tween-20, 1% NP-40,150 mM KCl, 1× proteinase inhibitors) and sonicated with a Hielscher UP200S sonicator for 3 × 5 min on ice using 100% amplitude and 0,5 s/0,5 s intervals. To ensure even fragmentation, 5 mM CaCl_2_ and 30 U micrococcal nuclease were added and the lysates incubated for 30 min at room temperature, after which the digestion was stopped with 20 mM EGTA. The fragmentation of DNA to an average size of 500 bp was confirmed through agarose electrophoresis (Supplementary Figure S1) and aliquots containing 3 mg mitochondrial protein used for immunoprecipitation.

The lysate was cleared by rotation with 50 μl non-coupled magnetic agarose beads (Chromotec #bmab-20) for 30 min at 4°C, after which the beads were removed. For flag-IP, 40 μl anti-flag magnetic beads (Origene #TA150042) were added to the lysate and rotated at 4°C overnight. The beads were washed three times with RIPA buffer (10 mM Tris pH 7,4, 150 mM NaCl, 1 mM EDTA, 1% NP-40, 0.5% deoxycholate, 0.1% SDS, proteinase inhibitors) and once with 1× PBS, after which the crosslinking was reversed in 100 μl 1× PBS + 20 μg Proteinase K at 65°C overnight. After removal of the beads DNA was extracted from the samples using the peqGold blood and tissue DNA mini kit (VWR) and mtDNA fragments were quantified by SybrGreen quantitative PCR using the following primers:

D-loop: HSmtDNA37F (AGC TCT CCA TGC ATT TGG) and HSmtDNA269R (GGA AAG TGG CTG TGC AGA CA); 16S: HSmtDNA2279R (TCA CCC TAT AGA AGA ACT AAT G) and HSmtDNA2400R (GTT GGT TGA TTG TAG ATA TTG G); ND1: HSmtDNA3322F (CTC CTA CTC CTC ATT GTA) and HSmtDNA3410R (TTG CGT AGT TGT ATA TAG C); ND3: HSmtDNA10131F (ACC ACA ACT CAA CGG CTA CA) and HSmtDNA10382R (TGT AGT CA TCA TAG GCC AGA C); ND5: HSmtDNA13463F (GCA GCC TAG CAT TAG CAG GA) and HSmtDNA13593R (TGT CAG GGA GGT AGC GAT GA).

### Mapping of linearized ends by nanopore sequencing

The coordinates of mtDNA break points was determined using mtDNA isolated from HEK293 T-REx cells expressing mtTop3α or mtTop3α-Y362F as well as empty vector control cells. 5 μg mtDNA were treated with 20 U RNase I for 6 h at 37°C to remove RNA and linearized with NheI, a restriction enzyme cutting human mtDNA once at position 4,581. The linearized mtDNA sequencing libraries were prepared using the PCR-free, Ligation Sequencing Kit (SQK-LSK109, Oxford Nanopore Technologies, UK) along with the Native Barcoding Expansion (EXP-NBD104, Oxford Nanopore Technologies, UK) following the manufacturers protocol. The barcoded libraries were then sequenced on a MinION Flongle flowcell. Demultiplexing, basecalling and removal of barcodes and adapters was done offline using Guppy version 4.2.2 (Oxford Nanopore Technologies, 2020). The sequencing reads were mapped to the human mtDNA sequence (NC_012920) with the first basepair set as 4,581 (*Nhe*l restriction cut site) using minimap2 with the setting -x map-ont (28) and alignments were output in PAF format.

### RNA probe preparation and detection

For detection with strand-specific probes the nts 16 177–40 of human mtDNA were cloned into the pCR2.1 TOPO vector in both orientations using a TOPO cloning kit (Invitrogen, P/N 46-0801). The plasmids were linearized using *Bam*HI and 1μg of DNA used for *in vitro* transcription with a DIG RNA labeling mix (Roche, 11277073910) and T7 RNA polymerase (Thermo Scientific, EP0111) for 2 hours in 37°C. The reaction was stopped by addition of 2 μl of 0.2 M EDTA and the heat-denatured reaction mix used to hybridize Southern blot membranes overnight in Church's buffer (0.25 M Na-phosphate pH 7.2, 7% SDS, 1 mM EDTA). The blots were washed 3 × 30 min in wash buffer (1× SSC, 0.1% SDS) and blocked in 75 mM maleic acid, 200mM NaCl pH 7.5, 5 % skimmed milk powder for 1 hour in room temperature. The membranes were incubated with Anti-Digoxigenin-AP Fab fragment antibody (1:10 000) (Roche, 11093274910) for 2 h in room temperature, washed 2 × 15 min with blocking solution + 0.3 % Tween and further washed for 5 min with wash buffer (100 mM Tris–HCl pH 9.5, 100 mM NaCl). The signal was detected with CDP-Star chemiluminescence substrate (1:100) (Roche, 11685627001).

## RESULTS

### Knockdown of Top3α affects mtDNA maintenance

Previous studies of both Top1mt and the two isoforms of Top2 have indicated that these enzymes regulate the general topology of mtDNA and thus indirectly also the initiation of replication. In contrast, there is no compelling evidence that these enzymes would be directly involved in the relief of topological tension around the progressing replication fork. Thus, we decided to investigate whether the mitochondrial Top3α topoisomerase might be essential for replication fork progression and DNA synthesis. For this purpose, we studied changes in mtDNA replication caused by depletion of Top3α using a range of electrophoretic analyses.

**Figure 1.**
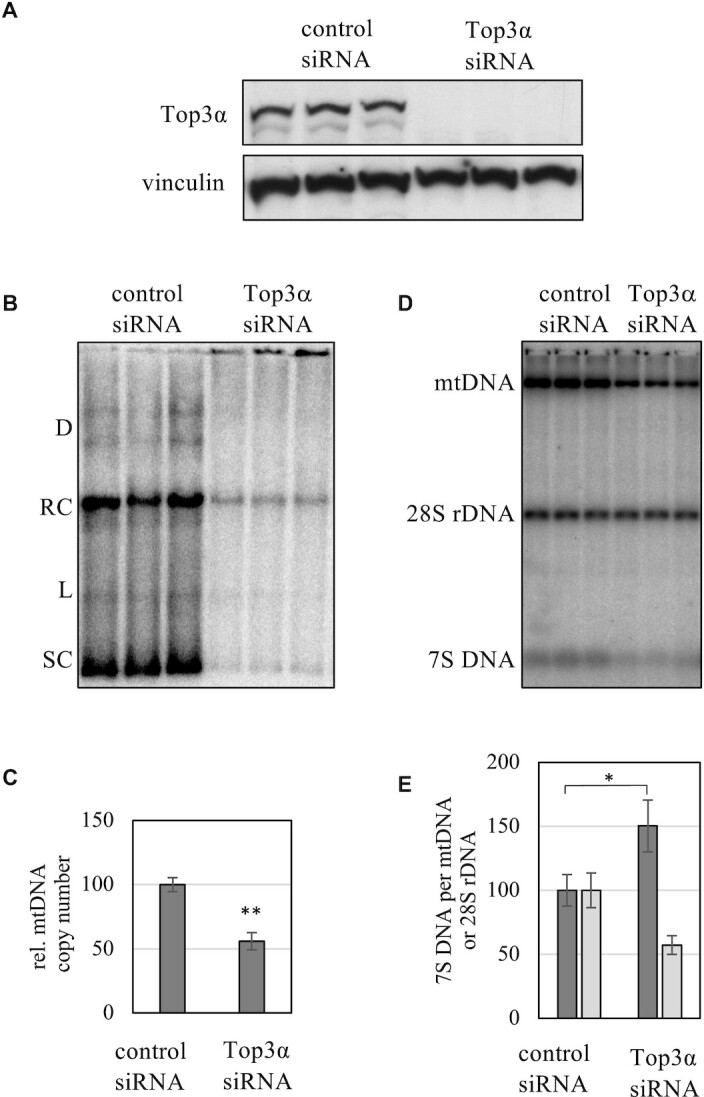
Effects of Top3α knockdown on mtDNA in HeLa cells transfected transiently with either siRNA against Top3α or scrambled control siRNA. (**A**) Reduction of Top3α protein levels after 6 days of siRNA treatment. Vinculin as loading control. (**B**) Analysis of mtDNA topology. Knockdown of Top3α protein for 6 days does reduce mtDNA levels but does not alter mtDNA topology. D – dimers; RC – relaxed monomeric circles; L – linearized mtDNA; SC - supercoiled mtDNA (**C**) Quantification of the relative mtDNA copy number depletion after 6 days of knockdown (*n* = 3, *P* < 0.01 with Student's *t*-test). (**D**) Analysis of 7S DNA, mtDNA and 28S gene levels in *Hind*III-digested, heat-treated total DNA of knockdown and control cells. (**E**) Quantification of the ratio of 7S to mtDNA (dark grey bars) and 28S rDNA (light grey bars). A steep loss of 7S DNA is observed, but the ratio of 7S DNA per mtDNA rises.

The transient knockdown of Top3α by siRNA lead to clear changes in mtDNA structure and abundance (Figure 1). Upon loss of Top3α (Figure 1A) mtDNA levels dropped to ca. 50% after 6 days (Figure 1 B and C). Interestingly, despite this clear depletion, no effect on the proportions of the various topological forms of single mtDNA molecules was observed, suggesting that Top3α is not essential for the regulation of overall mtDNA conformation (Figure 1B, for explanation of the various topological forms Supplementary Figure S2). We also observed the proportional increase of high molecular weight aggregates of mtDNA described previously (13), confirming the proposed role of Top3α in mtDNA segregation. Interestingly, the loss of Top3α causes a steep decrease of both mtDNA and 7S DNA, but an increase in the ratio of 7S to mtDNA (Figure 1D and E). 7S DNA, the short third strand of DNA associated with the non-coding region of the mtDNA, is assumed to be produced by frequent mtDNA replication starting at O_H_ and being aborted at the so-called termination associated sequence TAS (29). While the reason for the high synthesis and turnover rate of this short replication product is not understood, it can serve as an indicator for disturbances of mtDNA replication. The increase of 7S DNA per mtDNA template might be a compensatory reaction to the observed mtDNA depletion or a sign of impaired extension.

**Figure 2.**
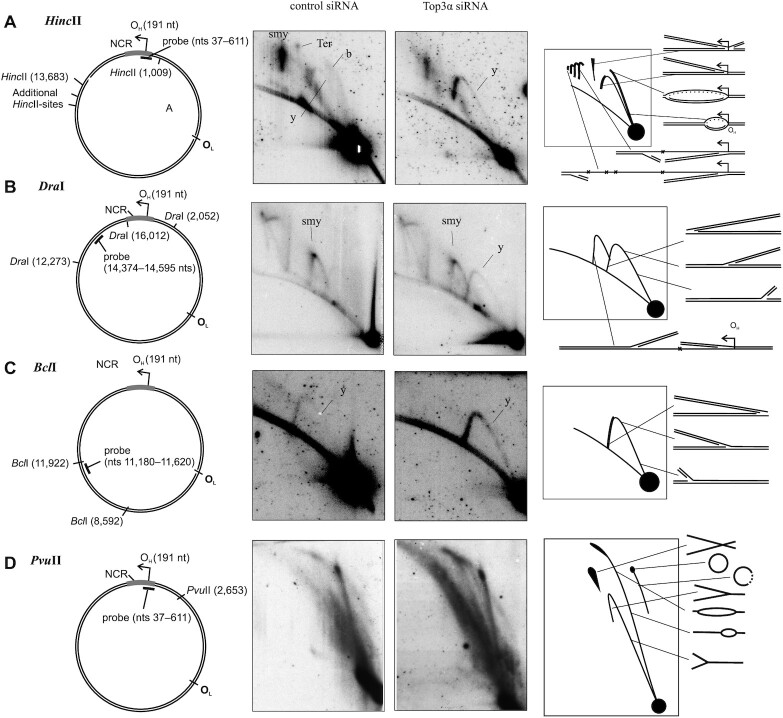
Analysis of replication processes in Top3α knockdown and control cells. The replication patterns of mitochondrial DNA in HeLa cells after knockdown and control treatment analysed by 2D-AGE of total cellular DNA as well as schematic representations of the used digests and the observed types of replication intermediates. (**A**) A digest with *Hinc*II shows the non-coding region including the O_H_ area of initiation and termination. After Top3α knockdown, intermediates indicative of asynchronous replication and termination intermediates decrease and a bubble arc is not observed anymore, while the y-shaped molecules get more abundant. (**B**) A *Dra*I digest showing a fragment downstream of OH visualizes two y-arcs, the left slow-moving one indicative of asynchronous replication. Top3α knockdown shifts the patterns slightly to the fully-double-stranded right y-arc but does not lead to a visible bubble arc that would suggest replication initiation in this region. (**C**) A *Bcl*I digest probed for the fragment containing the ND4 gene shows enrichment of y-shaped replication intermediates upon Top3α knockdown, but no bubble-shaped intermediates. (**D**) A digest with *Pvu*II, cutting human mtDNA once, gives an overview over the whole mtDNA molecule, indicating an enrichment of replication intermediates and thus stalling, as well as a small proportion of synchronous replication upon Top3α knockdown, visible as y-shaped molecules. Ter – termination intermediates; y – y-arc; b – bubble arc; smy – slow moving y-arc.

To elucidate which effects Top3α knockdown had on the mtDNA replication process we analyzed the abundance and structure of replication intermediates using neutral/neutral two-dimensional agarose gel electrophoresis (2D-AGE, Figure 2) (25). The same method was used by Nicholls *et al.* (13), and the presented results suggest replication stalling to occur upon loss of mtTop3α, but the authors interpreted the observed stalling to be a consequence of the impaired segregation of the replicated molecules. HeLa cells with reduced Top3α protein levels show reduced levels of asynchronous replication and accumulation of fully double-stranded replication intermediates, indicative of replication stalling. Replication stalling was observed not only in the non-coding region harbouring replication initiation and termination (HincII digest, Figure 2A), but also in all other studied areas, including the *Dra*I fragment covering the first stretch of asynchronous replication (Figure 2B), the ND4 region (BclI digest, Figure 2C), the AccI fragment containing O_L_ (Supplementary Figure S3A) as well as in PvuII (Figure 2D) and BamHI (Supplementary Figure S3B) digests showing the replication pattern of the whole mitochondrial DNA molecule. Top3α knockdown strongly reduced replication already in its early stages as evident from the complete lack of detectable replication bubbles in the HincII fragment containing the main replication origin O_H_. Instead, accumulation of y-shaped molecules containing single replication forks, that are indicative of replication stalling beyond the initiation loci, was evident in all regions, suggesting that replication fork progression is impaired throughout the replication process. While the majority of replication intermediates arose through asynchronous replication, visible as bubble arc in the PvuII digest and as slow-moving y-arc in the DraI digest (2B and D), upon Top3α knockdown, additionally a small proportion of y-shaped molecules were observed in the PvuII analysis, suggesting that the induced stalling induced a partial switch of replication mode towards synchronous strand-coupled replication. This was confirmed by the analysis of fork directionality, showing that upon Top3a knockdown forks move through the *ND6/CYTB* region in both directions (Supplementary Figure S4).

### Overexpression of mtTop3α suppresses mtDNA replication

Replicative topoisomerases remove the negative supercoils accumulating behind the replication machinery as well as the positive supercoils ahead of the replication machinery, relieving the torsional tension on the molecule and allowing the replication to progress. As the observed negative effects of Top3α knockdown on replication fork progression could indicate that Top3α has a similar role in mitochondria, we aimed to test this hypothesis by employing the overexpression of mitochondrial Top3α as a tool to enhance its activity.

**Figure 3.**
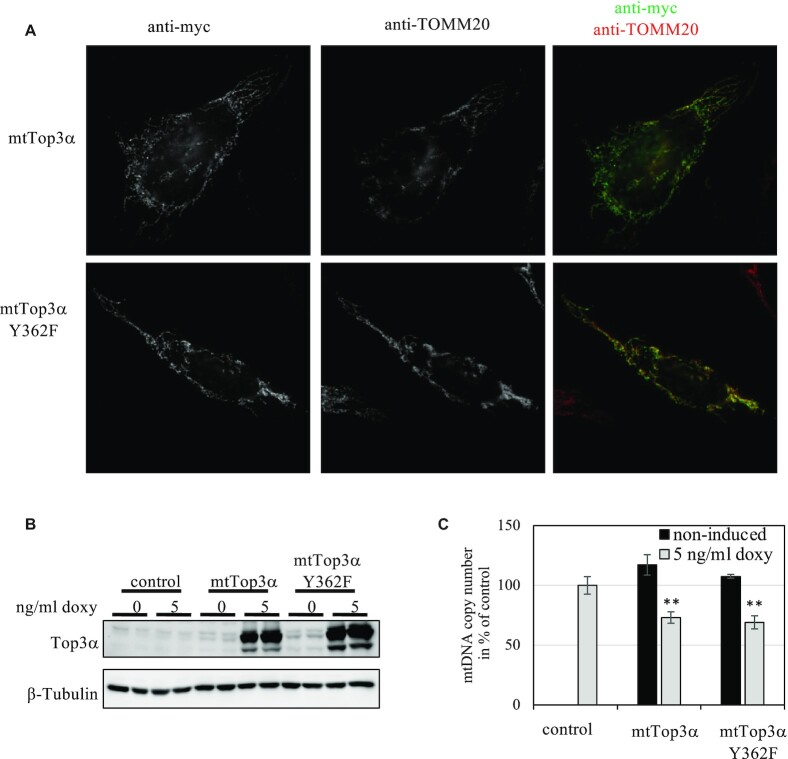
Ectopic expression of mitochondrial Top3α constructs. (**A**) Verification of the mitochondrial localization of mtTop3α constructs by immunocytochemistry in transiently transfected HeLa cells. The ectopically expressed proteins were visualized using a mouse-anti-myc antibody detecting the C-terminal tag and donkey-anti-mouse-Alexa488 secondary antibody, while mitochondria were stained with a rabbit-anti-TOMM20 antibody and a secondary donkey-anti-rabbit-Alexa594 antibody. Both the catalytic active and inactive variant of mtTop3α are clearly localized to mitochondria. (**B**) Overexpression of mitochondrially targeted Top3α constructs in stable HEK293 T-REx cells induced with 5 ng/ml doxycycline. HEK293 T-REx cells carrying only the empty pcDNA5 FRT/TO vector serve as control. (**C**) Effects of mtTop3α overexpression on mtDNA levels. Expression of both active and inactive mtTop3α lead to a 25% decrease in mtDNA copy number compared to control after 2 days of induction (*n* = 3, *P* < 0.01, ANOVA with Tukey post-hoc analysis). black bars: no induction, grey bars: expression induced with 5 ng/ml doxycycline.

Expression of a Top3α gene construct with the mitochondrial targeting sequence and mutated nuclear start codon did not increase total cellular Top3α protein noticeably. Instead, clear overexpression of the protein was achieved only when we also mutated the nuclear localization signal at the C-terminus, creating a strictly mitochondrial version of Top3α (mtTop3α). We also created a catalytically inactive, but otherwise identical version of the same mitochondrial construct (mtTop3α-Y362F) by replacement of the catalytic tyrosine. As the DNA-binding capacity of this enzyme version is unaltered, it served as a control to identify adverse effects caused by the artificial overexpression of a DNA-binding protein. We confirmed the proteins to be exclusively localized in mitochondria using immunocytochemistry, finding convincing co-localization with TOMM20, a mitochondrial marker protein (Figure 3A). Induced overexpression of both mtTop3α variants in HEK293 T-REx cells caused an expression level correlating to the amount of doxycycline (Figure 3B and Supplementary Figure S5), and 5 μg/ml doxycycline increased total cellular Top3α protein levels ca. 10-fold compared to empty vector controls (Figure 3B). Using this inducible expression system, we found that low levels of mtTop3α caused by the leakiness of the expression system mildly increased mtDNA copy number, while induced expression of high levels of mtTop3α caused a significant depletion of mtDNA (Figure 3C). The catalytic inactive mtTop3α-Y362F variant instead did not influence mtDNA without induction, but also caused depletion upon strong overexpression (Figure 3C). The observed depletion of mtDNA affected the mitochondrial network only little, leading to mild fragmentation of the mitochondrial network, but no obvious alterations in mtDNA nucleoid structure (Supplementary Figure S6). The observed effects were caused exclusively by overexpression of the mitochondrially targeted mtTop3α, while a strictly nuclear version of Top3α did not have any effect on mtDNA copy number, topology or 7S DNA levels (Supplementary Figure S7).

**Figure 4.**
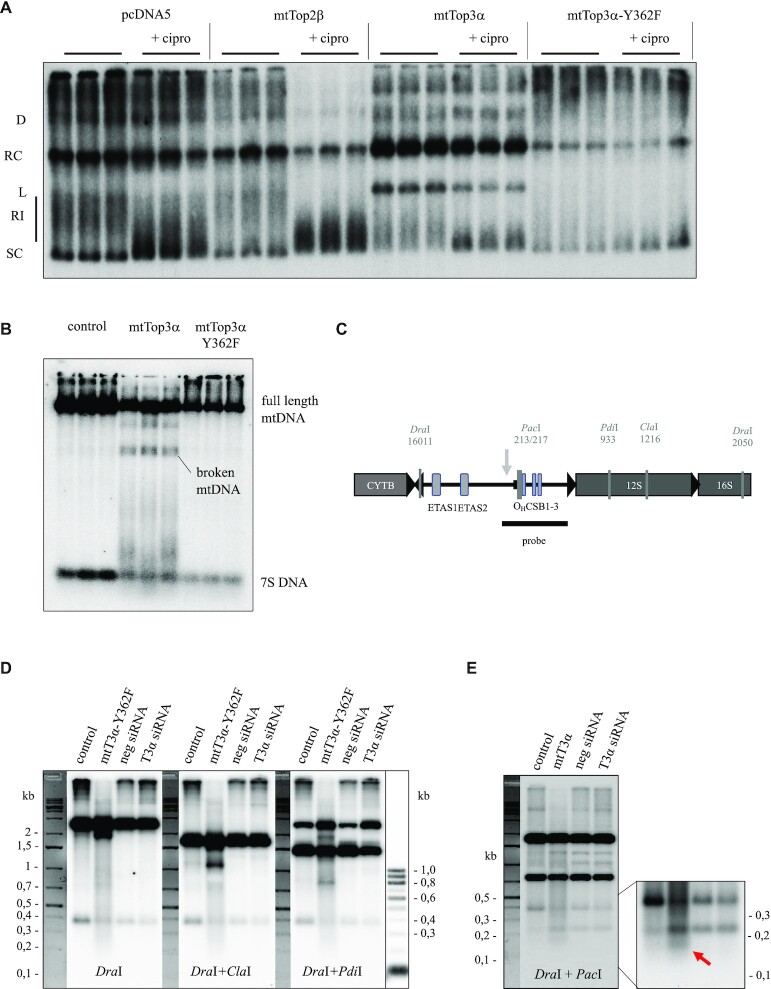
Effects of mtTop3α overexpression on mtDNA structure. (**A**) Analysis of mtDNA topology changes upon overexpression of mitochondrial Top3α variants (48 h 5 ng/ml doxycycline). D – dimers; RC – relaxed monomeric circles; L – linearized mtDNA; SC – supercoiled mtDNA; RI – replication intermediates. (**B**) Analysis of 7S DNA levels and DNA breakage in *Hind*III-linearized mtDNA upon mtTop3α overexpression (24 h 5 ng/ml doxycycline) (**C**) Schematic representation of the restriction sites in human mtDNA used to map the mtDNA breakpoint in mtTop3α-overexpressing cells and the location of the breakpoint as determined in the following panels. (**D** and **E**) Digestion pattern of mtDNA from control and mtTop3α-overexpressing HEK293 T-REx cells induced with 5 ng/ml doxycycline as well as siRNA control and Top3α knockdown cells. Total DNA was digested with *Dra*I and, where indicated, additionally with the indicated restriction enzymes, separated over a 0.5% gel and probed with with nts 40–617 of mtDNA. In mtTop3α-overexpressing cells additional bands of ∼2 kb (*Dra*I digest), ∼1.2 kb (DraI + ClaI), ∼800 bp (DraI + PdiI) and ∼150 bp are observed (marked with an arrow in the stronger exposed zoom area in E).

We studied the reasons for mtDNA depletion upon mtTop3α expression in more detail using mtDNA topology analysis (Figure 4A and Supplementary Figure S2), comparing the findings to both control cells and mtTop3α-Y362F-expressing cells. To distinguish the functions of Top3α from other mitochondrial topoisomerases, cells overexpressing mitochondrially targeted Top2β were analyzed, as well as the effect of the type II topoisomerase inhibitor ciprofloxacin added to the growth medium at a concentration of 80 μg/ml during the second half of the induction time.

As described previously, ciprofloxacin treatment did not cause any mtDNA breaks, but resulted in a strong accumulation of supercoiled mtDNA and reduced the signal between supercoiled and linear DNA band, where e.g. replicating molecules migrate (8). Overexpression of mtTop2β alone did not alter mtDNA topology itself but exaggerated the impact of ciprofloxacin. Interestingly, the loss of replicating molecules was accompanied by a reduction in multimeric mtDNA forms, with the exception of a low signal from dimers, supporting the idea that mtDNA catenation and replication are coupled. Overexpression of mtTop3α reduced the abundance of supercoiled mtDNA in favor of relaxed circles, but also caused a dramatic increase in linearized mtDNA. The signal of non-migrating mtDNA in the slot of the gel as well as between supercoiled and relaxed dimeric circles was clearly reduced compared to controls, indicating enhanced decatenation as shown previously (13). Ciprofloxacin did not alter this decrease in catenation, but slightly reduced the levels of broken molecules, probably due to its inhibition of replication initiation (8). The catalytically inactive mtTop3α-Y362F reduced the mtDNA levels overall, but did not cause mtDNA linearization. Instead, it acted in a dominant-negative fashion, with overexpression leading to a strong accumulation of high molecular weight forms as reported for knockdown of Top3α (13). Ciprofloxacin addition altered the topological distribution only marginally, suggesting that replication is nearly absent in mtTop3α-Y362F-expressing cells.

As a type I topoisomerase, mtTop3α catalyzes single-strand breaks, and thus the appearance of linearized mtDNA upon overexpression was surprising. We analyzed the break points of these molecules using one-dimensional gel electrophoresis of HindIII-digested DNA, and found a defined band of ca. 6 kb, indicating a homogeneous break point in the non-coding region (Figure 4B). The precise location of this double-strand break was mapped by a series of restriction digests (Figure 4C–E). While the location of the breakpoint in the non-coding area ca. 60 bp downstream of O_H_ was confirmed, no precise coordinates could be determined by this approach.

The mapping of the end coordinates of the linearized mtDNA using nanopore sequencing technology did not provide any further insight, and no precise breakpoint could be identified (Supplementary Figure S8). In all samples, breaks were far more abundant in the non-coding region than in the coding part of mtDNA, and the majority of ends locate around the start and end of 7S DNA around O_H_ and before the TAS. Interestingly, there was no difference in cells overexpressing mtTop3α compared to control or mtTop3α-Y362F overexpressors, indicating that the resulting ends are unsuitable for blunt-end ligation even after an end repair step, e.g. due to large overhangs of one strand. Top3α is a type I topoisomerase introducing only single-strand cuts, but the introduction of such single-strand cuts in the vicinity of a pre-existing nick within the non-coding region might lead to linearization of mtDNA in the non-coding region. Overexpression of mtTop3α or mtTop3α-Y362F reduced 7S levels but caused no significant change in the ratio of 7S to mtDNA (Figure 4B and Supplementary Figure S9).

Interestingly, we also observed the accumulation of linear mtDNA molecules longer than 7S DNA in cells expressing mtTop3α, suggesting that replication forks stall and break in the early phase of replication. The observed DNA species ranged in size from 500 to ca. 1500 nucleotides and were single-stranded H-strand sequences, as indicated by their sensitivity to S1 nuclease and their hybridization with an L-strand, but not an H-strand probe (Supplementary Figure S10). Combined with the reduction in mtDNA copy number, this phenomenon suggests that an excess in active Top3α strongly impairs the progression of the replication machinery, causing replication fork stalling soon after initiation and the release of the newly synthetized H-strand. Although high expression of the mtTop3α-Y362F variant depleted 7S levels and mtDNA levels, it did not cause strand breaks or release of H-strand molecules, indicating that the catalytic activity of mtTop3α is required for this phenomenon.

**Figure 5.**
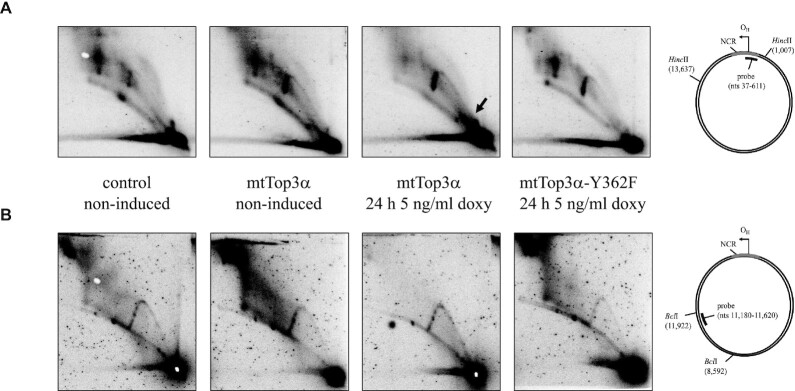
Changes of replication processes upon mtTop3α overexpression. (**A**) Analysis of the *Hinc*II fragment containing the non-coding region of mtDNA with initiation and termination sites. (**B**) Replication progression in the *Bcl*I fragment in the coxIII/ND4 region. While the leaky expression in non-induced T-Rex mtTop3α cells induces an increase in smy and termination intermediates, indicating acceleration of asynchronous replication, the strong overexpression of mtTop3α upon doxycycline induction causes loss of smy-arcs, a slight increase in double-stranded intermediates and accumulation of replication intermediates in early stages of replication (marked by an arrow in A). The mtDNA of cells overexpressing mtTop3α-Y362F shows nearly normal replication. A detailed interpretation guide of the 2D panels can be found in Figure 2 and in the text.

This hypothesis was confirmed by 2D-AGE analysis of replication intermediates (Figure 5).

HEK293 T-REx cells carrying the empty pcDNA5 FRT/TO showed the replication intermediate pattern typical for normal proliferating cells, a strand-asynchronous mode involving the replication of the lagging strand with a considerable delay compared to the leading strand (30). In the non-coding region, this asynchronous replication is visualized by a blunt and broad bubble arc, indicative of partly single-stranded molecules containing a replication bubble (Figure 5A). Additional indications of this asynchronous replication mechanism are a detectable, but not very strong y-arc (Y) as well as a cloud of non-linear molecules with high molecular weight, which arise from a restriction site block by single-strandedness or RNA hybridization and form the so-called slow-moving y-arcs (smy). As termination of replication occurs in the same region, this process is also observed through its intermediate molecules, consisting of double-y-shaped molecules with two approaching replication forks (ter). In the region containing the ND4 gene only y-shaped molecules with a single replication fork are observed (Figure 5B).

Non-induced HEK293 T-REx mtTop3α cells showed a rather similar pattern, with only slightly reduced bubble arc intensity but increased abundance of smy's. This indicates the low-level leaky expression of the transgene is not disturbing mtDNA replication, but instead increases the progression of the replication fork and thus shifts the ratio of H-strand synthesis vs. maturation of the asynchronous replication intermediates. The analysis of the ND4 region using BclI as restriction digest confirms these findings.

High level expression of mtTop3α strongly reduced smy's and caused a sharpened initiation bubble arc, a tell-tale sign of replication fork slow-down. Additionally, it caused an accumulation of replication intermediates in the region directly downstream of O_H_ (marked by an arrow in Figure 5A), indicating replication stalling soon after initiation. Under the same condition, the region containing the ND4 gene did show complete absence of slow-moving replication intermediates, but no change in double-stranded intermediates, suggesting no replication forks to move into this area anymore (Figure 5B). The termination intermediates, abundant in undisturbed mtDNA replication, were reduced upon high mtTop3α expression, which could be a direct result of the increased resolution activity of Top3α or an indirect consequence of slowed-down replication. We did not observe any sign of enhanced initiation outside of the noncoding region, which would indicate a switch in replication mode towards strand-coupled replication (31).

In contrast, the expression of mtTop3α-Y362F altered the general replication pattern only mildly, suggesting that DNA-binding alone is not sufficient to cause the alterations observed upon mtTop3α expression, although in the ND4 fragment a reduction of replication intermediates was observed.

### Top3α interacts with the mitochondrial replication fork

**Figure 6.**
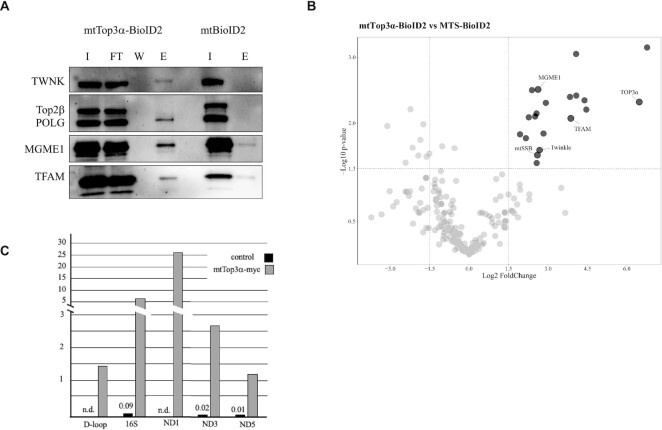
Analysis of Top3α interaction with mtDNA and nucleoid proteins. (**A**) Western blot analysis of proteins labelled by mtTop3α -BioID2 and purified by streptavidin affinity. HEK293 T-REx mtTop3α BioID2-HA cells were induced for 24h with 3 ng/ml doxycycline to reach low level expression of the mtTop3α-BioID2 fusion protein. The medium was changed to biotin-containing, doxycycline-free medium for another 24h, after which mitochondria were extracted. Biotinylated proteins were purified using streptavidin affinity beads and analyzed by western blot. I – input lysate; FT – flowthrough (unbound proteins); W – last wash; E – elution. (**B**) Mass Spectrometric analysis of proteins enriched in streptavidin affinity purification after labelling with mtTop3α-BioID2 bait (*n* = 3) compared with that of MTS-BioID2 control bait (*n* = 3). The red points on the volcano plot indicate the 20 proteins considered to be significantly enriched in mtTop3α-BioID2 purification as delimited by p-value < 0.05 and log2FC $ \ge$ 1.5. The mitochondrial nucleoid proteins TFAM, mtSSB, Twinkle, MGME1 as well as Top3a are highlighted. The x-axis represents fold change of triplicates’ means on log_2_ scale, the y-axis represents the negative log_10_-transformed *P*-value calculated in unpaired two-sample Student's *t*-test. (**C**) Quantification of mtDNA regions enriched by Chromatin immunoprecipitation of myc-tagged mtTop3α. mtTop3α-myc was pulled down from crosslinked mitochondrial lysates after 12 h of low-level expression (induction with 1 ng/ml doxycycline) using myc-trap beads and co-purified mtDNA fragments were quantified by quantitative PCR. Mitochondrial lysate from mtTop3α-flag-expressing cells served as a negative control.

To interrogate the role of Top3α in mtDNA replication progression in more detail, we studied the interaction of mitochondrial Top3α with known mitochondrial DNA maintenance proteins using BioID2 labeling. In this technique the protein of interest is fused to a small biotin ligase, allowing the *in vivo* labeling and subsequent isolation of proteins in immediate proximity to the bait (32). To avoid artefacts, the mtTop3α-BioID2 fusion protein was expressed only at low level for 24 h, followed by a 24 h labeling phase without further expression. Using streptavidin affinity purification, we found the mitochondrial DNA helicase TWNK to be biotinylated by mtTop3α-BioID2, but not by a mitochondrially targeted BioID2 alone (Figure 6A). The catalytic subunit of the replicative DNA polymerase Pol γ (POLG) was specifically enriched with mtTop3α-BioID2, verifying that mtTop3α is located in the immediate vicinity of the mitochondrial DNA replication fork. Top2β in contrast was not biotinylated, indicating that it is not in close contact with mtTop3α. Other known mtDNA interactors MGME1 and TFAM instead were enriched upon mtTop3α-BioID expression, although a weak signal was also detected in the mtBioID2 control.

To verify these findings and to identify other proteins in close vicinity to the mitochondrial Top3α enzyme, we analyzed the purified biotinylated proteins by mass spectrometry and peptide mass fingerprinting. Besides Top3α itself, only 19 proteins were significantly enriched in mtTop3α-BioID2 purifications, among them were TWNK, mtSSB and TFAM, but also MGME1 (Figure 6B and Table 1). Only three of these proteins are not listed in MitoCarta 3.0. The low number of identified proteins and the presence of known mtDNA interactors among them indicates the specificity of our approach and the close vicinity required for efficient biotinylation. Using co-immunoprecipitation, we did not find evidence for a direct physical interaction of mtTop3α with TWNK or POLG (data not shown), which is in line with existing data for other topoisomerases involved in torsional stress release during replication (33,34).

To confirm the interaction of Top3α with mtDNA also outside of the non-coding region, we performed a chromatin immunoprecipitation experiment, using 293TREx cells expressing low levels of either a myc- or a flag-tagged mtTop3α gene. mtTop3α was pulled down from crosslinked mitochondrial lysates using anti-myc-antibody beads and the concentration of various mtDNA sequences in the pulldown determined by quantitative PCR. All five mtDNA sequences measured were strongly enriched in the myc-tagged version compared to the flag-tagged lysate serving as control, indicating that mtTop3α binds to mtDNA also outside of the non-coding region (Figure 6C).

### Manipulation of Top3α influences transcription

**Figure 7.**
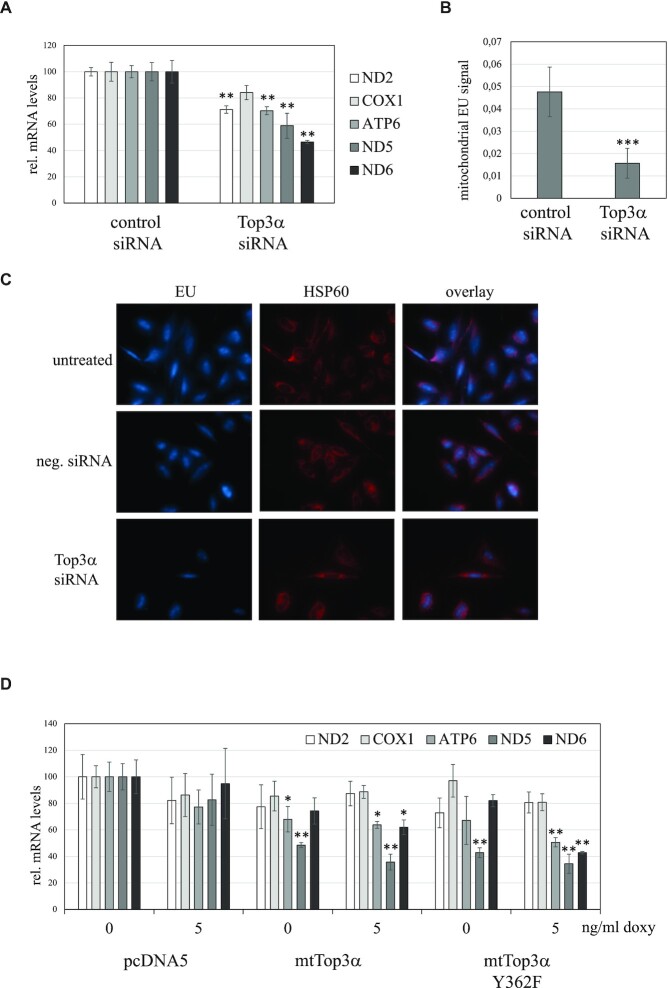
Top3α effects on transcription. (**A**) Steady-state mRNA levels of ND2 and ND5 after 6 days of Top3α knockdown in HeLa cells. All measured mRNA levels expect COX1 decreased (Statistical analysis with ANOVA/Tukey post-hoc analysis, *n* = 3; *P* < 0.01 for ND2 vs control, *P* < 0,01 for ATP6 vs control, *P* < 0,01 for ND5 vs control, *P* < 0.01 for ND6 versus control). (**B**) Quantification of nascent RNA labelling in mitochondria of HeLa cells upon knockdown of mtTop3α. Freshy synthetized RNA was labelled with EU for 90 min and visualized by immunocytochemistry, using an antibody against HSP60 as mitochondrial marker. The EU signal the whole cell as well as mitochondria is reduced upon loss of mtTop3α, indicating a reduced transcription rate. *P* < 0.001 with student's t-test. (**C**) Examples of the EU/HSP60 stainings used for the quantification in (B). (**D**) Steady-state transcript levels in HEK293 T-REx cells without induction and after 24 h of 5 ng/ml doxycycline. Cells containing the empty vector pcDNA5 FRT/TO served as a control. A clear decrease in ND5 mRNA levels was observed even with background expression of both catalytic active and inactive Top3α, also ND6 levels decreased in induced cell lines, while ND2 and COX1 stayed unaltered. Statistical analysis with ANOVA/Tukey post-hoc analysis; *n* = 3, **P < 0.01, *P < 0.05 for non-induced and induced mtTop3α and mtTop3α-Y362F versus the respective control cells.

As Top3α might release the topological tension during replication, but also during transcription, we analyzed the effects of Top3α knockdown and overexpression on the steady-state levels of mitochondrial transcripts. Knockdown of Top3α decreased the level of mitochondrial mRNAs (Figure 7A). The most affected transcripts were ND5 and ND6, fitting with the idea that in the absence of Top3α torsional stress impairs the progression of the RNA polymerase, leading to reduced transcription of downstream genes on the H-strand. The reduction of mitochondrial transcripts was caused by a decreased transcription rate, as EU-labelling of nascent transcripts showed a reduction in RNA synthesis (Figure 7B, C and Supplementary Figure S11).

The overexpression of both mtTop3α and mtTop3α-Y362F caused a reduction in ATP6, ND5 and ND6 mRNA levels, but not in ND2 and COXI transcripts (Figure 7D). The fact that both the catalytically active and inactive form of Top3α modify the expression profile of mitochondrial transcripts in a similar fashion suggests that this effect is caused indirectly through abundant DNA-binding or the stalling effects on replication, and not by a specific function during mtDNA transcription. As only downstream transcripts are affected, the disrupting effect appears to be caused by a problem in progression of the transcription machinery rather than in transcription initiation.

Overexpression of mtTop3α, regardless of the catalytic activity, did not impact early transcripts (ND2 and COXI), but reduced ND5 transcript levels dramatically. Interestingly, even the low-level expression in non-induced mtTop3α and mtTop3α Y362F cells was sufficient to decrease ND5 steady-state levels to half, while high-level expression was required for an impact on ND6, that is transcribed from the L-strand.

## DISCUSSION

**Figure 8.**
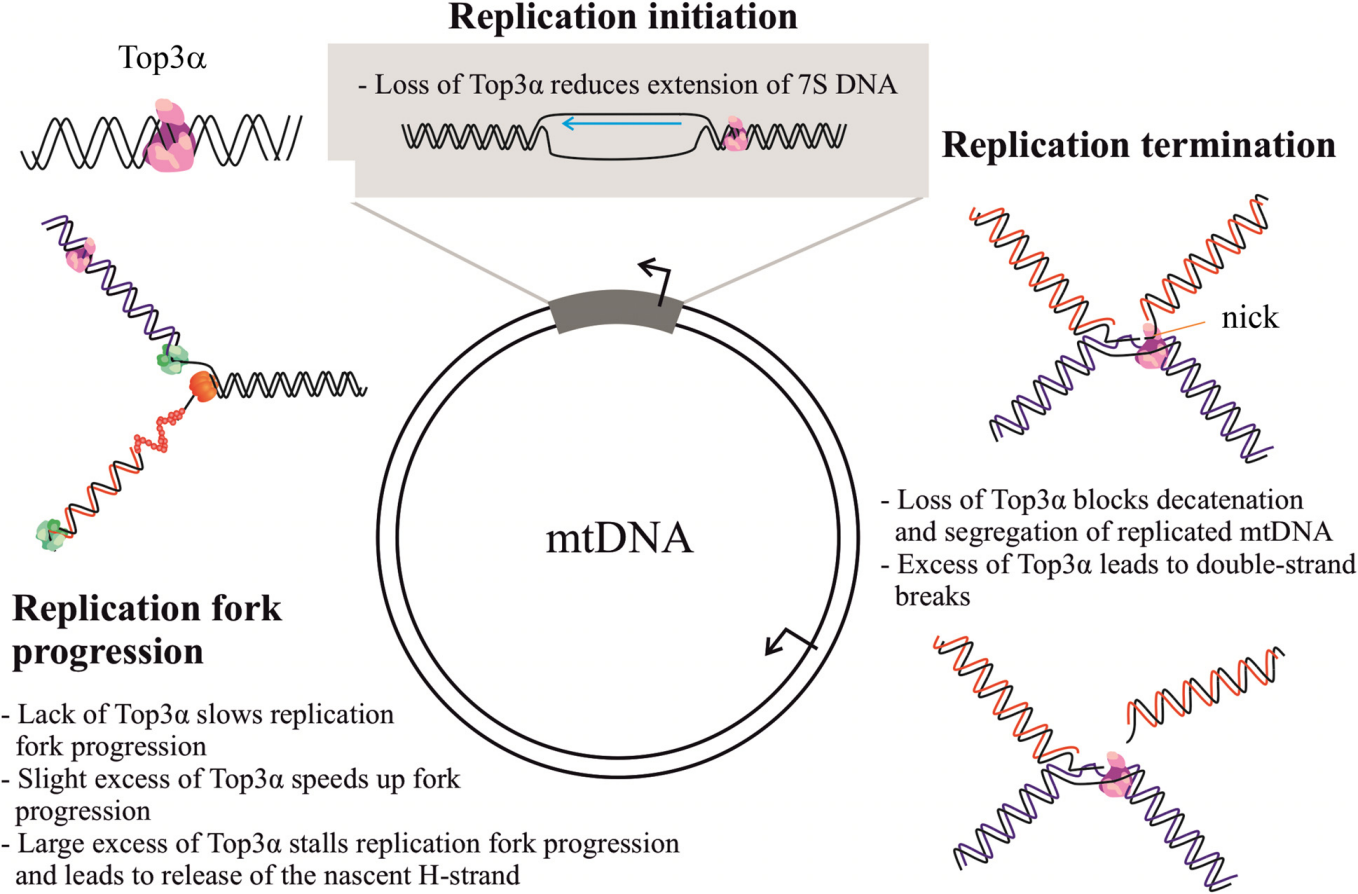
Schematic representation of the roles of Top3α in mtDNA replication.

In this study, we found evidence for multiple functions of Top3α in mtDNA replication: mtTop3α localizes close to the mitochondrial replication fork, and knockdown of the protein leads to clear replication stalling, indicating its direct action at the replication fork (Figure 8). As a type IA topoisomerase, Top3α can catalyze the relaxation of negative, but not positive supercoils, suggesting it relieves the negative torsional stress accumulating after the replication fork. Still, the positive supercoiling formed ahead of the replication fork requires a topoisomerase for removal, and while this is catalyzed by gyrase and topoisomerase IV in bacteria (35), in mitochondria this function might be fulfilled by Top1mt or Top2β. Alternatively, the positive tension can spread beyond the replication fork, inducing coiling of the daughter strands, so-called precatenanes. In *E. coli*, these precatenanes are removed by TopIII, a topoisomerase related to eukaryotic Top3α (33), and a similar function of Top3α in mitochondria is possible.

Depletion of Top3α reduces replication intermediates containing an initiation bubble, thus also the initiation phase of mtDNA replication (i.e. the extension of 7S DNA beyond the TAS sequence) is dependent on Top3α activity. As no change in general mtDNA topology was observed, Top3α appears to influence replication initiation locally, e.g. by the relaxation of D-loop structures. A function in the region downstream of O_H_ would explain the abundant creation of double-strand breaks we observed in this area upon mtTop3α overexpression, as single-strand breaks introduced by Top3α together with pre-existing nicks could cause the linearization of the mtDNA molecules.

While we observed replicating stalling upon both knockdown and overexpression of mtTop3α, we found the minority of replication intermediates to stem from synchronous replication, that has been previously observed in many cases of replication stalling (19,22,36). This is likely due to the strong reduction of replication fork progression upon near complete loss of Top3α, that impairs the extension of replication bubbles regardless of the initiation location.

Finally, we confirmed the role of Top3α during the last steps of replication, when the two daughter molecules need to be separated to allow segregation during mitochondrial fission. As Top3α is not a typical decatenase with its ability to catalyze single-strand breaks, we propose it to act before both DNA strands have been fully replicated. The termination intermediates enriched at the end of mtDNA replication contain two approaching replication forks, but also a non-replicated sequence separating them. Top3α might decatenate the daughter molecules before the newly synthesized strands are ligated, thus requiring only a single strand cut for segregation.

Replication and transcription of mtDNA are interacting in many ways: On one hand, mitochondrial transcript availability has been found to limit strand-asynchronous replication and to induce a switch to synchronous, bidirectional replication (36). On the other hand, a stalled replication fork poses a roadblock for transcription, and collisions of replication and transcription machineries are considered highly dangerous for genome integrity (37,38). Our data does not suggest transcript limitation to cause the observed replication stalling upon loss of mtTop3α, as steady-state transcript levels are still more than 50%, when replication stalling is observed, replication stalling is observed in the area of ND2 and coxI with barely impaired transcript levels, and nascent transcripts are still produced, leading to a detectable EU staining. Instead, fork collision, as a consequence of replication stalling, might cause the observed impairment of transcription even without a direct involvement of Top3α in transcription.

While Top3α has been speculated to play a role in recombination of mtDNA, where the cooperation of a topoisomerase and a helicase could facilitate strand exchange reactions, we did not find any indication for the involvement of Top3α in this process in mitochondria. Although mtTop3α overexpression raised the levels of linearized mtDNA dramatically, a situation previously found to increase mtDNA recombination (19), we did not observe enhanced x-spikes or other signs of recombination in this study.

Taken together, our findings show that Top3α is essential for replication fork progression in mtDNA replication and influences transcription rates. However, future work might elucidate additional roles of this versatile enzyme in mtDNA maintenance.

## DATA AVAILABILITY

The data and materials used in this study are available from the corresponding author upon reasonable request. Proteomics data has been deposited to PRIDE under project number PXD035416 (https://www.ebi.ac.uk/pride/).

## Supplementary Material

gkac660_Supplemental_Files
