# Supplementary material for: Top3α is the replicative topoisomerase in mitochondrial DNA replication
Source: Nucleic Acids Res. 2022 Jul 29;50(15):8733–48. doi: 10.1093/nar/gkac660 (PMC9410902; doi:10.1093/nar/gkac660)
Supplement: gkac660_Supplemental_Files [file gkac660_supplemental_files.zip › supplementary figures S1-S11.pdf]

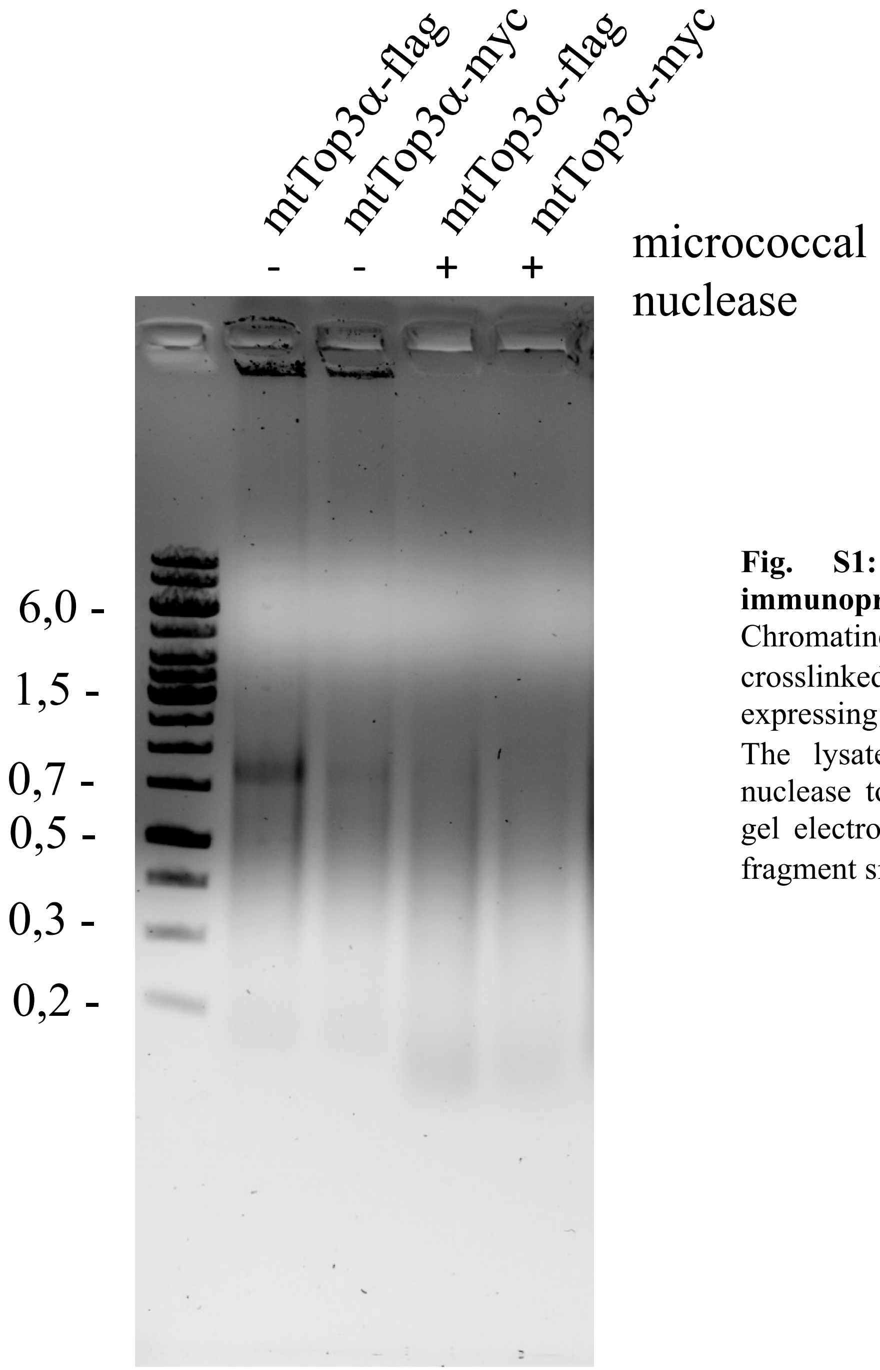

Fig. S1: Fragmentation of mtDNA in Chromatin immunoprecipitation samples

Chromatine immunorepcipitation was performed using crosslinked mitochondrial lysates from 293 TREx cells expressing either mtTop3α-flag or mtTop3α -myc.

The lysates were sonicated and digested with micrococcal nuclease to ensure uniform fragmentation of mtDNA. Agarose gel electrophoresis of deproteinated lysates showed an average fragment size of 500 bp.

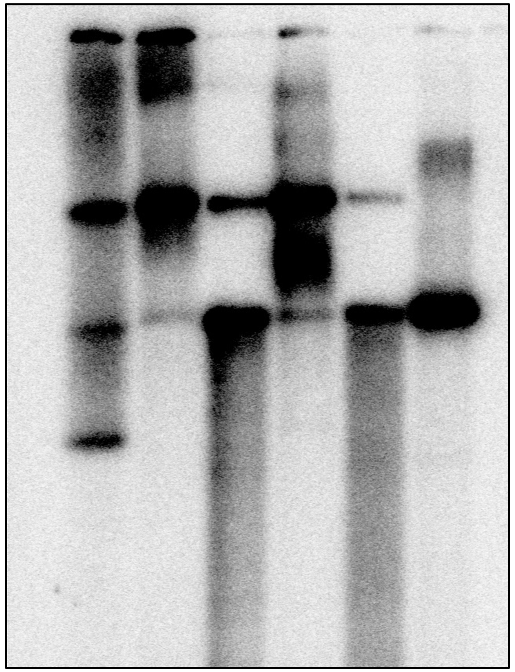

Fig. S2: Topology of mtDNA.
DNA of HEK293 cells was treated *in vitro* with different topology-modifying enzymes, separated over a 0.4 % agarose/TBE gel and probed with nts 13,456-13,777 of mtDNA.
In untreated mtDNA, supercoiled and relaxed circles, linearized mtDNA and high molecular weight forms of mtDNA are observed. Treatment with *E. coli* Topoisomerase I relaxes both monomeric and multimeric supercoiled forms. T7 endonuclease cuts cruciform DNA structures and removes all high molecular weight forms except truly dimeric mtDNA, but also introduces nicks and strand breaks into monomeric mtDNA. Topoisomerase IV decatenates concatemers of mtDNA, revealing true dimers in relaxed and supercoiled form. *BamH*I cuts mtDNA once, converting all topological forms to linears.

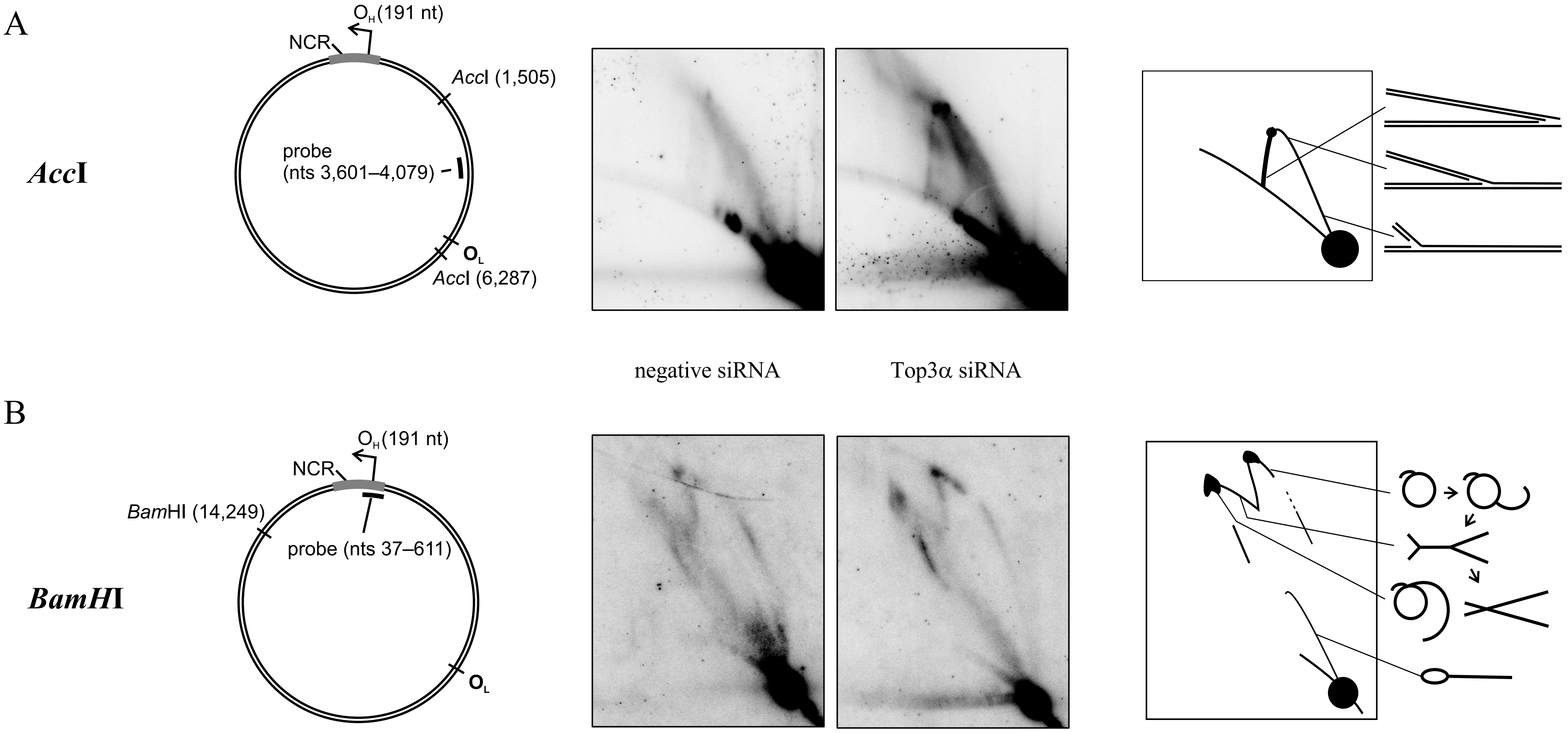

Fig. S3: mtDNA replication pattern after knockdown of Top3α.
DNA of HeLa cells 6 days after transfection with either negative control siRNA or siRNA against Top3α was analyzed by 2D-NAGE. A) *Acc*I digest of control and knockdown cells. The blot was probed with nts 3,601-4,079 of mtDNA, thus showing the fragment containing O_L. The knockdown of Top3α caused accumulation of y-shaped replication intermediates and pronounced pausing at the IQM tRNA cluster, while the transfection with negative siRNA did not change the replication pattern compared to untreated controls. B) Analysis of the whole mtDNA molecule by *BamH*I, a restriction enzyme cutting mtDNA once in the *ND6* gene. As in the AccI digest, also in this fragment accumulation of replication intermediates is observed. The impaired resolution upon loss of Top3α leads to an enhancement of x-shaped molecules, visible as diagonal streak in both controls and knockdown cells.

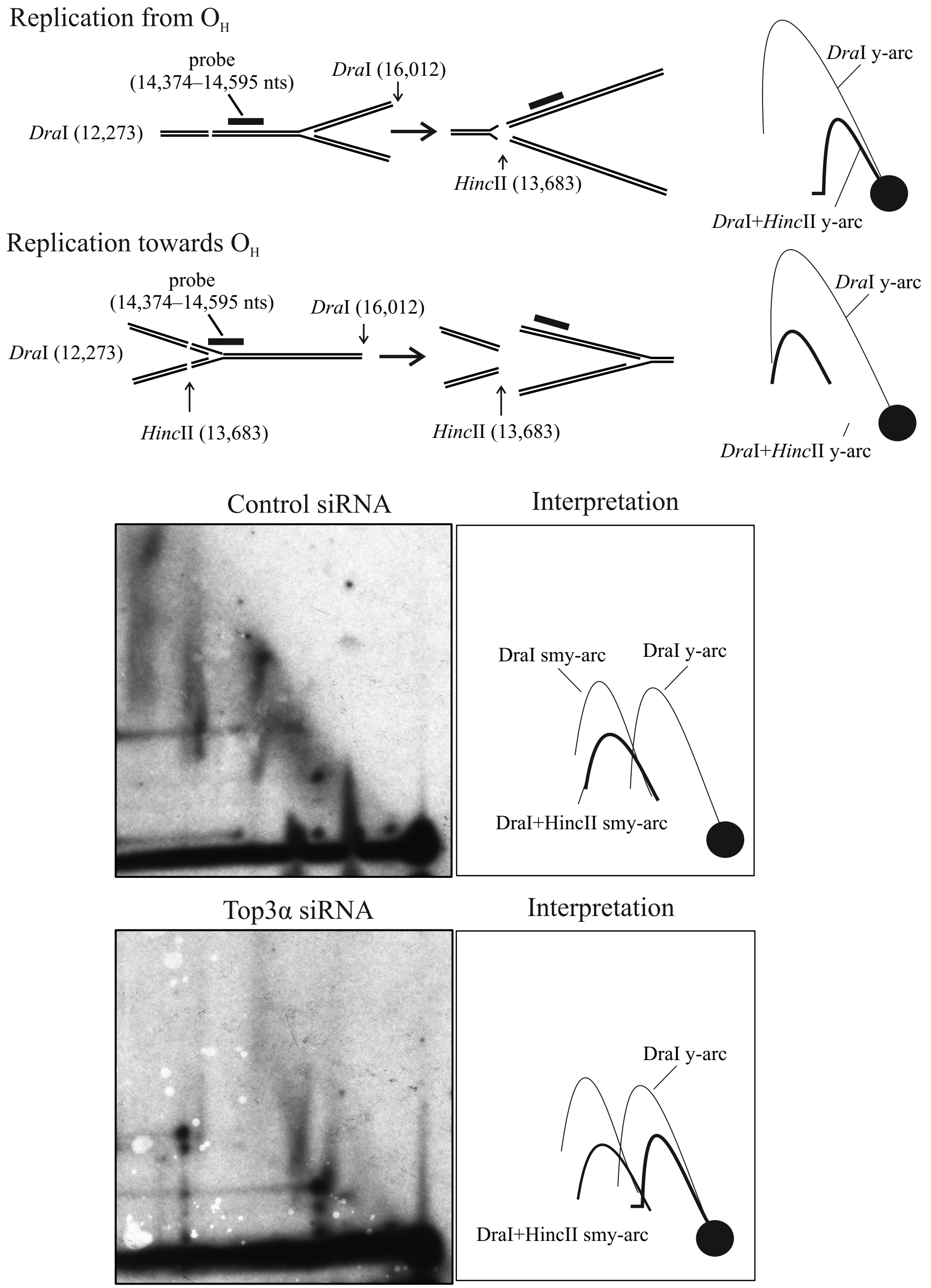

Fig. S4: Directionality of the replication fork in Top3α knockdown and control cells.

DNA of HeLa cells 6 days after transfection with either negative control siRNA or siRNA against Top3α was digested with *Dra*I, separated over the first dimension, in-gel digested with *Hinc*II and separated over the second dimension. The trimming of the replication intermediates before the second dimension allows to determine in which direction the replication fork passes through the observed fragment. The majority of replication intermediates are incompletely digested as typical for asynchronous replication and thus form a slow-moving y-arc (smy, Fig. 2), while a faint regular y-arc consisting of synchronous replication intermediates is barely visible. After the *Hinc*II digest, control cells show a clear depressed smy-arc (thick line in the interpretation sketch), indicating replication to proceed mainly from O_H. Cells experiencing knockdown of Top3α show both a faint depressed smy as well as a very faint depressed y-arc, having thus low levels of replication proceeding in both directions.

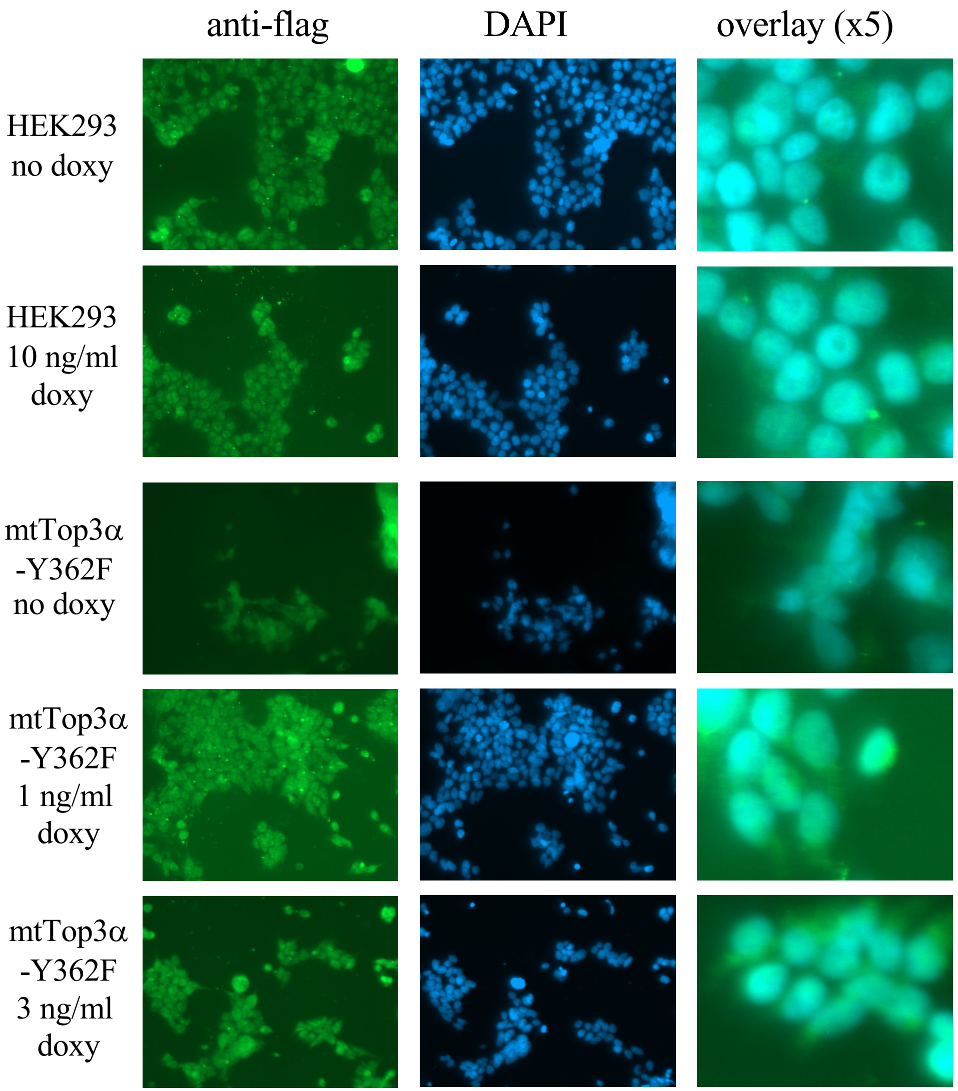

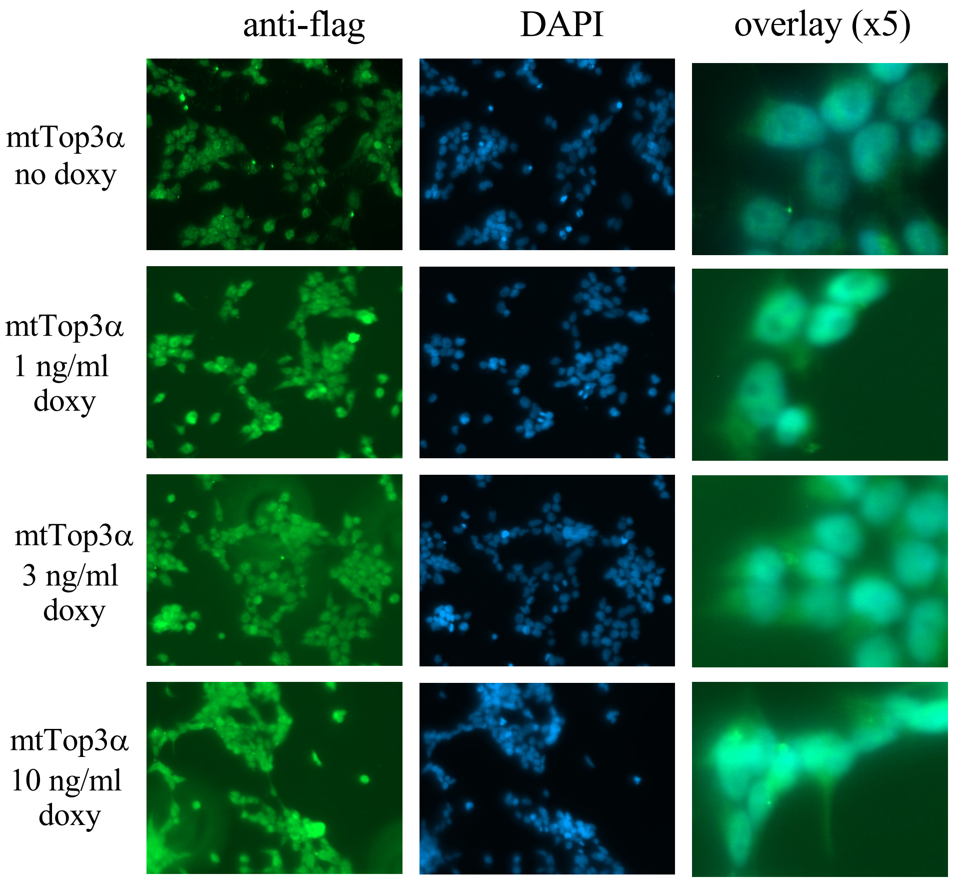

Figure S5: Immunocytochemistry of HEK293 T-REx cell lines after 24 h induction with increasing doxycycline concrentrations. mtTop3α and mtTop3α-Y362F were stained using an anti-flag antibody and an anti-rabbit Alexa488 secondary antibody, while the nuclei were stained with DAPI.

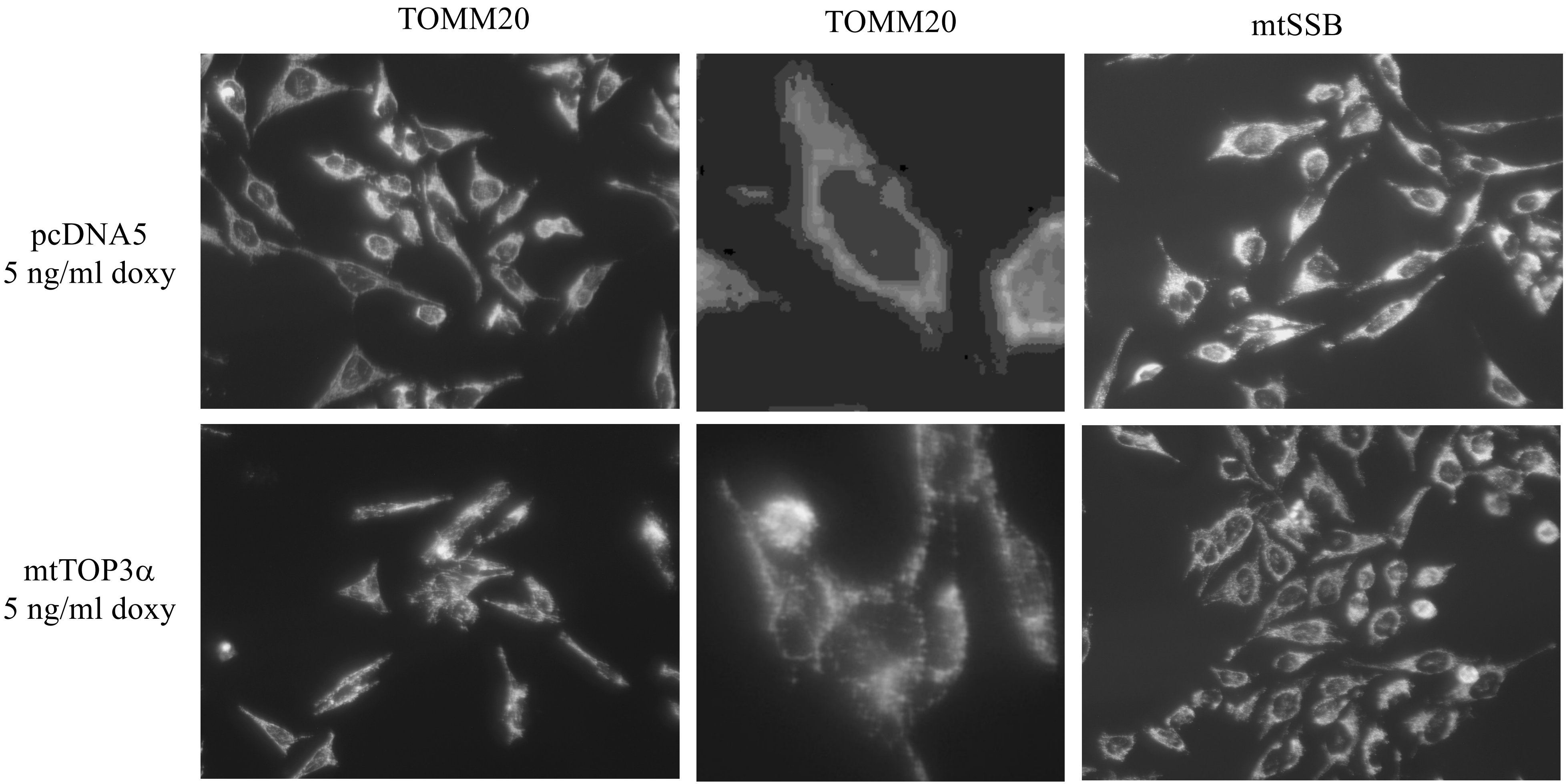

Fig. S6: Mitochondrial network and nucleoid integrity
HeLa T-REx mtTop3α cells and control cells containing only the empty pcDNA5 FRT/TO vector were induced for 24 h with 5 ng/ml doxycycline and after fixation immunostaining against the mitochodrial outer membrane protein TOMM20 and the mtDNA nucleoid component mtSSB was performed. While no alterations in nucleoids structure were observed, a mild fragmentation of the mitochondrial network was visible in mtTop3α-overexpressing cells.

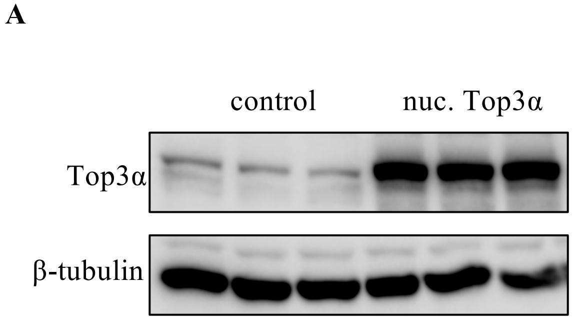

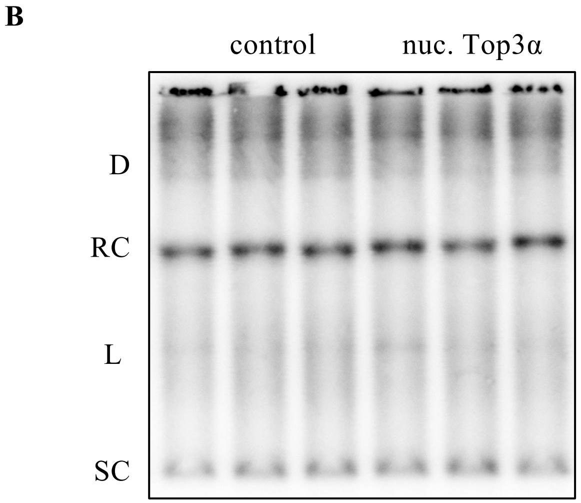

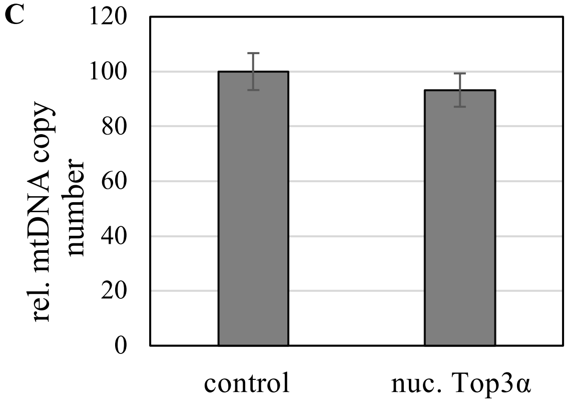

Fig. S7: **Effects of transient overexpression of nuclear Top3α in HEK293 cells.** A) Top3α levels were increased 2 days after transient transfection of nuclear version of Top3α lacking the mitochondrial targeting sequence. β-tubulin is used as loading control. B) mtDNA topology was not affected by overexpression of nuclear Top3α. C) No significant change was observed in the relative mtDNA copy number after transient nuclear Top3α overexpression.

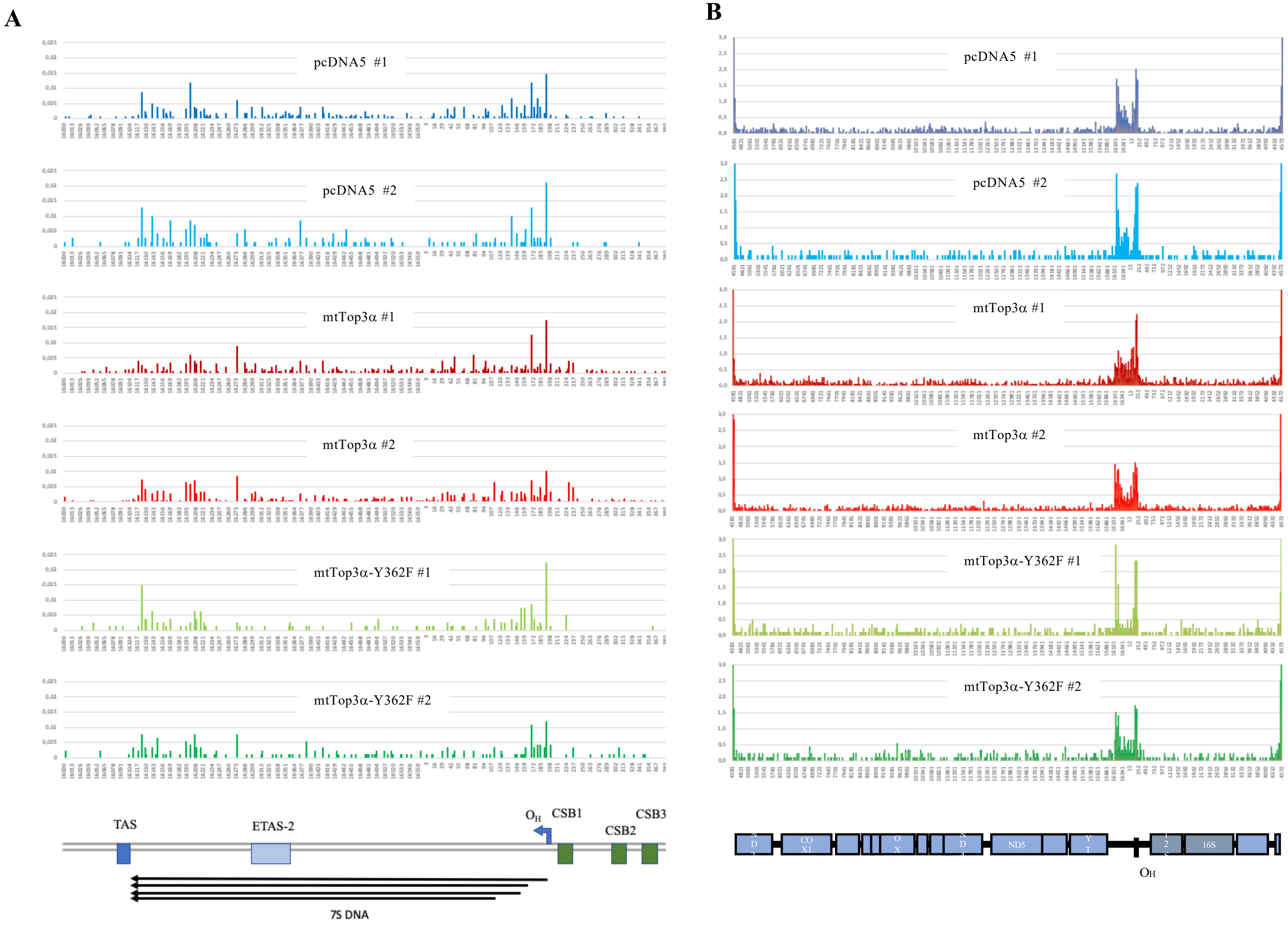

Figure S8: Mapping of linear mtDNA ends by nanopore sequencing.
mtDNA from HEK293 T-REx cells expressing mtTop3α and mtTop3α–Y362F for 2 days as well as control cells was linearized with *Nhe*I (cutting at position 4581 of human mtDNA), ligated to linkers and sequenced by nanopore technology. The resulting reads were aligned to human mtDNA and the end coordinates extracted. All samples showed a comparable pattern of DNA ends, with the majority of cuts localizing in the non-coding region between TAS and OH. No specific hotspot was detected in mtTop3α-overexpressing cells, suggesting that the resulting ends are not ligatable even after end repair and therefore not sequenced. A) Relative abundance of linearized ends in the non-coding region of mtDNA. B) Representation of ends in the whole mtDNA sequence. The y-axis is cropped at 3, so that the very high proportion of ends at the *Nhe*I restriction site at the extreme left and right of the x-axis is not visualized quantitatively.

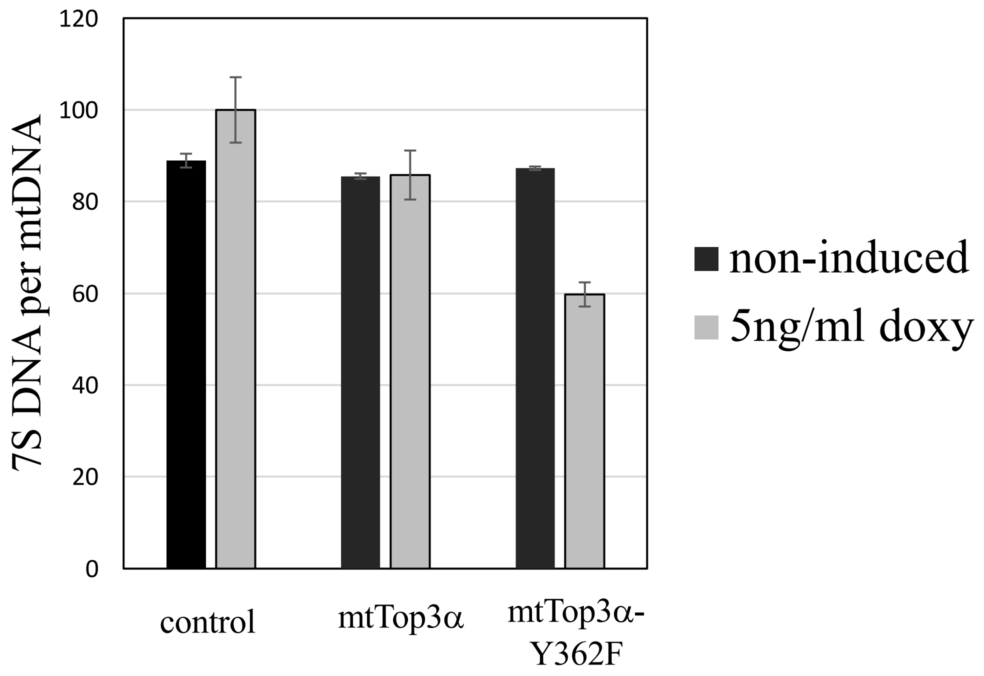

Fig. S9: Quantification of 7S levels in cells overexpressing mtTop3α
7S DNA levels per mtDNA in HeLa Trex cells carrying the empty pcDNA 5 vector, the mtTop3α or mtTop3α-Y632F and cultured for 48 h with or without 5 ng/ml doxycycline. No significant change in the ratio of 7S DNA to mtDNA was observed (ANOVA with post-hoc Tukey, $p>0.05$ for all conditions).

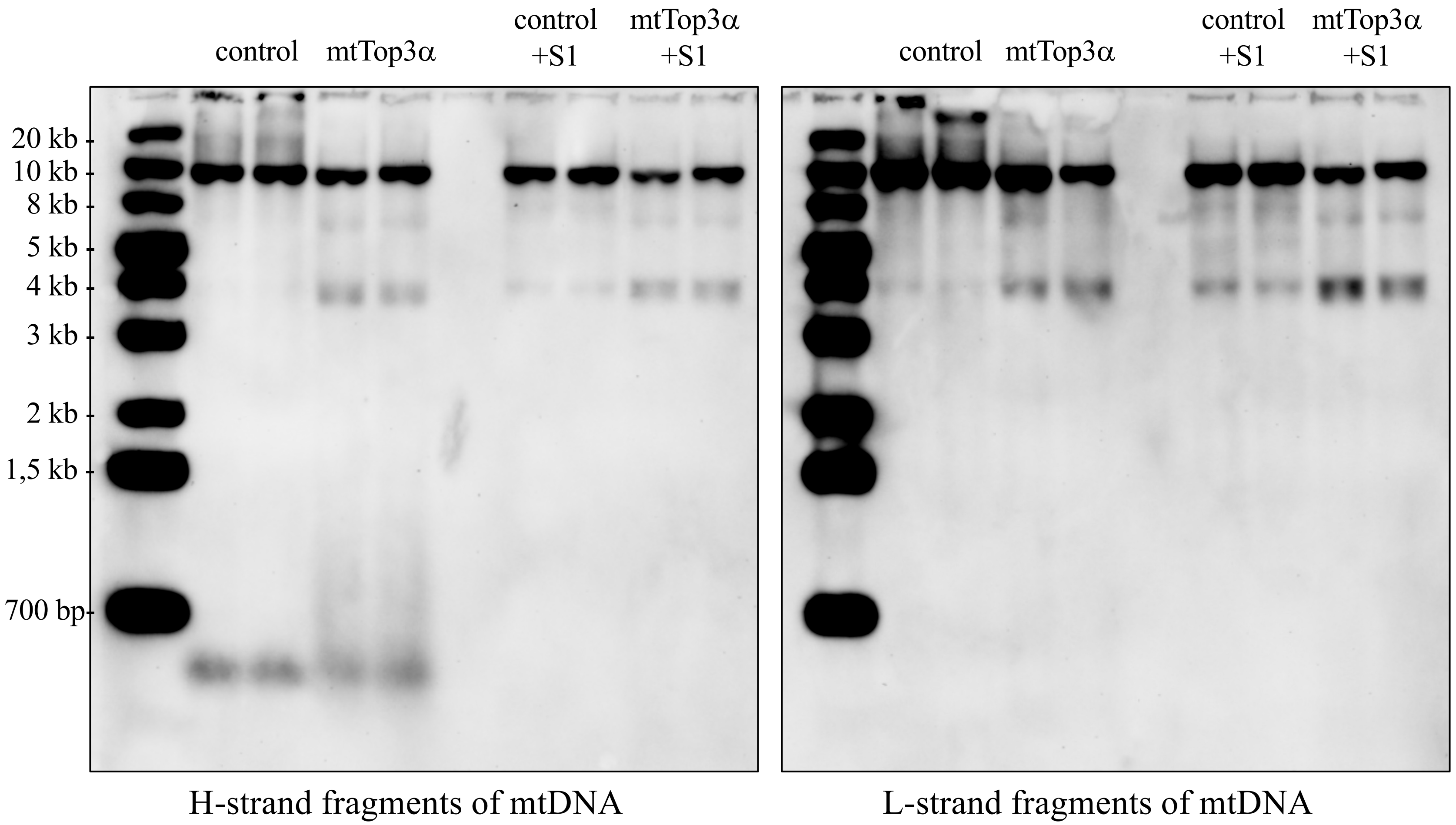

Fig. S10: S1 nuclease degrades the linearized mtDNA H-strand fragment caused by mtTop3α overexpression.
mtDNA of control and mtTop3a-overexpressing cells was digested with *Hind*II and S1-nuclease and analyzed by one-dimensional gel electrophoresis and Southern blot. Single-stranded probes with the sequence 16,177-40 of mtDNA were used for detection. HEK293 T-REx mtTop3α cells induced for 2 days with 5 ng/ml doxycycline show a clear accumulation of H-strand fragments of <2 kb size, while no such fragments are detected in control cells or with a probe recognizing the L-strand sequence. These fragments are degraded by *in vitro* treatment with S1 nuclease, an enzyme that degrades single-stranded nucleic acids, verifying their single-strand nature.

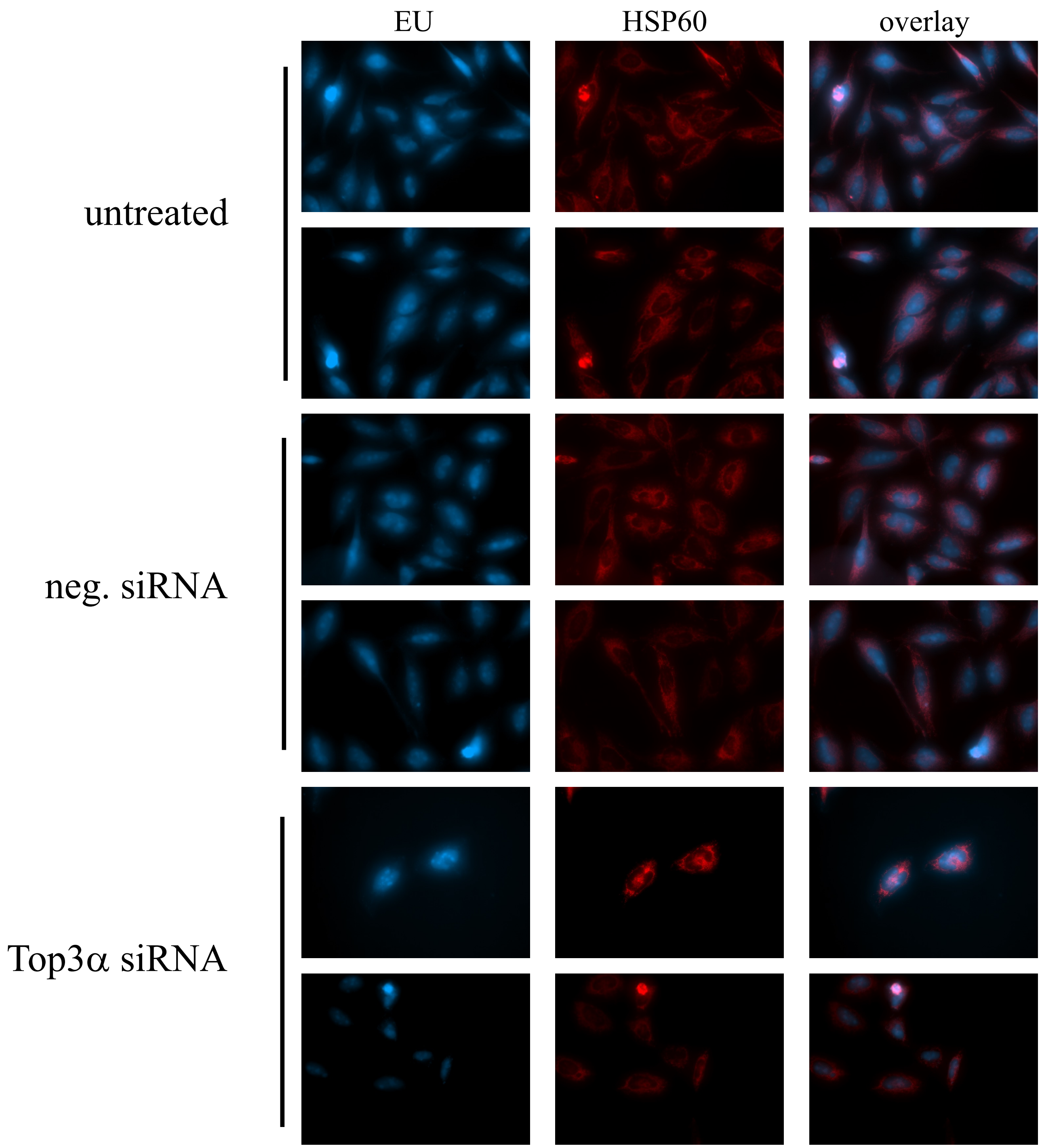

Fig. S11: nascent RNA immunocytochemistry after knockdown of Top3α

Nascent RNA labelling in HeLa cells upon knockdown of mtTop3α, as also shown in Fig. 7B. Freshy synthetized RNA was labelled with EU for 90 min, click-biotinylated and visualized by immunocytochemistry, using an antibody against HSP60 as mitochondrial marker. While the transfection of negative control siRNA did not affect the EU signal outside the nucleus, the loss of mtTop3α lead to a reduced labelling of cytosol and mitochondria, suggesting a reduced transcription rate.
